# Relation between Structure and Functionality in Photosynthetic Antenna Complex of Green Sulfur Bacteria: Efficiency under Natural Sunlight Pumping

**DOI:** 10.1021/acs.jpcb.6c00303

**Published:** 2026-05-15

**Authors:** Alessia Valzelli, Francesco Mattiotti, Jianshu Cao, Giuseppe Luca Celardo

**Affiliations:** † Dipartimento di Ingegneria dell’Informazione, 9300 Università degli Studi di Firenze, Firenze 50139, Italy; ‡ Dipartimento di Fisica e Astronomia, Università degli Studi di Firenze e CSDC, Sesto Fiorentino 50019, Italy; § Istituto Nazionale di Fisica Nucleare, Sezione di Firenze, Sesto Fiorentino 50019, Italy; ∥ Theoretische Physik, 9379 Universität des Saarlandes, Saarbrücken D-66123, Germany; ⊥ Department of Chemistry, 2167 Massachusetts Institute of Technology, 77 Massachusetts Avenue, Cambridge, Massachusetts 02139, United States; # European Laboratory for Non-Linear Spectroscopy (LENS), Universitá degli Studi di Firenze, Sesto Fiorentino 50019, Italy

## Abstract

Large-scale simulations of light–matter interaction in natural photosynthetic antenna complexes of the *Chlorobium tepidum* green sulfur bacteria (GSB), containing more than one hundred thousand chlorophyll molecules, comparable with natural size, have been performed. Here, we have modeled the entire process of exciton energy transfer, from sunlight absorption to exciton trapping in the reaction centers (RCs), in the presence of a thermal bath. The energy transfer has been analyzed using the radiative non-Hermitian Hamiltonian and by solving the rate equations for the populations. Sunlight pumping has been modeled as blackbody radiation at *T* = 5800 K, with an attenuation factor that takes the Sun–Earth distance into account. Cylindrical structures typical of GSB antenna complexes and the dimeric baseplate have been considered. The maximal antenna size, comparable to natural size, includes three adjacent, 4-walled concentric cylinders 1485.7 Å long, arranged over a dimeric baseplate with dimensions of 3075 Å × 1148 Å, for an overall number of molecules greater than 10^5^. Our analysis shows that under natural sunlight, in photosynthetic antennae of GSB, the number of excitations reaching the RC per unit time matches the RC closure rate, and the internal efficiency shows values close to ∼80%. We also considered cylindrical structures where the orientation of the dipoles does not reflect the natural one. Specifically, we vary continuously the angle of the transition dipole with respect to the cylinder’s main axis, focusing on the case where all dipoles are parallel to the cylinder axis. We also consider the important case where the dipoles are randomly oriented. In all cases, the light-harvesting efficiency is lower than in the natural structure, showing the high sensitivity of light harvesting to the specific orientation of the dipole moments. Our results provide a better understanding of the relationship between structure and functionality in natural photosynthetic antennae of green sulfur bacteria and could drive the design of efficient light-harvesting devices.

## Graphical abstract

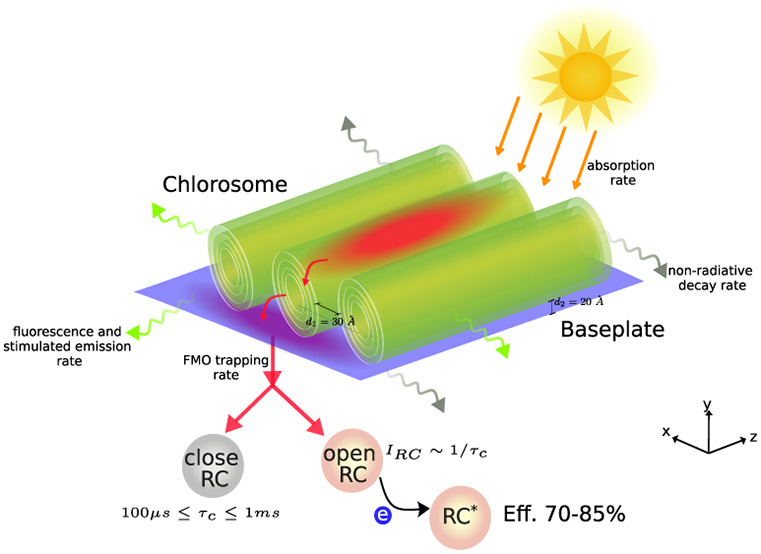

## 1 Introduction

Photosynthesis is a fundamental process able to capture sunlight and convert it into biochemical energy used to drive cellular processes.^1^ In this manuscript we model the process of sunlight absorption and energy transfer in the entire antenna complex of a species of photosynthetic anaerobic bacteria: the *Chlorobium tepidum* green sulfur bacteria (GSB). GSB use sunlight as their main source of energy. They are among the most efficient systems able to harvest sunlight in deep murky waters, where incident light levels are reduced much beyond the already dilute level on land.^2,3^ They are even able to perform photosynthesis with geothermal radiation from deep-sea hydrothermal vents at about 400 °C.^4^

The GSB antenna complexes are composed of a network of bacteriochlorophyll molecules (BChls *a*, *c*, *d* or *e*).^5^ In GSB BChl molecules are typically modeled as two-level systems (2LS). To each 2LS a transition dipole moment (TDM) is associated, which determines its coupling with both the electromagnetic field and other chlorophyll molecules. Photosynthesis in GSB involves chlorophyll pigments tightly packed in light-harvesting systems with (mostly) cylindrical shapes, known as chlorosomes. The geometry adopted for the GSB light-harvesting complexes is well-established in the literature. Specifically, the pigment organization and orientations within the GSB chlorosome have been extensively studied using infrared and resonance Raman spectroscopy, solid-state NMR, and cryo-EM. These studies reveal that pigments assemble into rod-like cylindrical aggregates characterized by lateral lamellae.^6–12^ In nature these structures typically range in size from 1000 to 2000 Å in length, with widths and depths varying between 600 and 1000 Å, and they can contain between 50000 and 250000 BChl *c*.^13–15^

Sunlight absorbed by the chlorosome is funneled to other complexes, such as the baseplate (BPL) and the Fenna–Matthews–Olson (FMO) trimer complexes, and finally to the reaction centers (RCs)^2,16^ where charge separation occurs, a process which precedes and drives all other photosynthetic steps.^2,3,17–22^ Once the RC receives an excitation, it produces a charge-separated state and electron transport through the RC begins. A reaction center is said to be in an open state before the excitation reaches it. Once an excitation reaches it and charge separation occurs, the RC goes in a closed state. The time needed for the RC to be open again is called the closure time (from 100 μs to few milliseconds.^23,24^ The closure time defines the frequency at which each RC is able to process an excitation.

Figure 1 shows the model we used to analyze the entire light-harvesting unit in GSB. The energy transfer process from the light-harvesting complex to the RC has a very high (near-80%) internal efficiency in these complexes.^16,25,26^ A possible origin of this incredible ability to utilize weak sources of incoherent light and funnel the collected energy to specific molecular aggregates could be brought back to the high level of symmetry and hierarchical organization characterizing the antenna complexes of bacterial photosynthetic organisms.^27,28^

**Figure 1:**
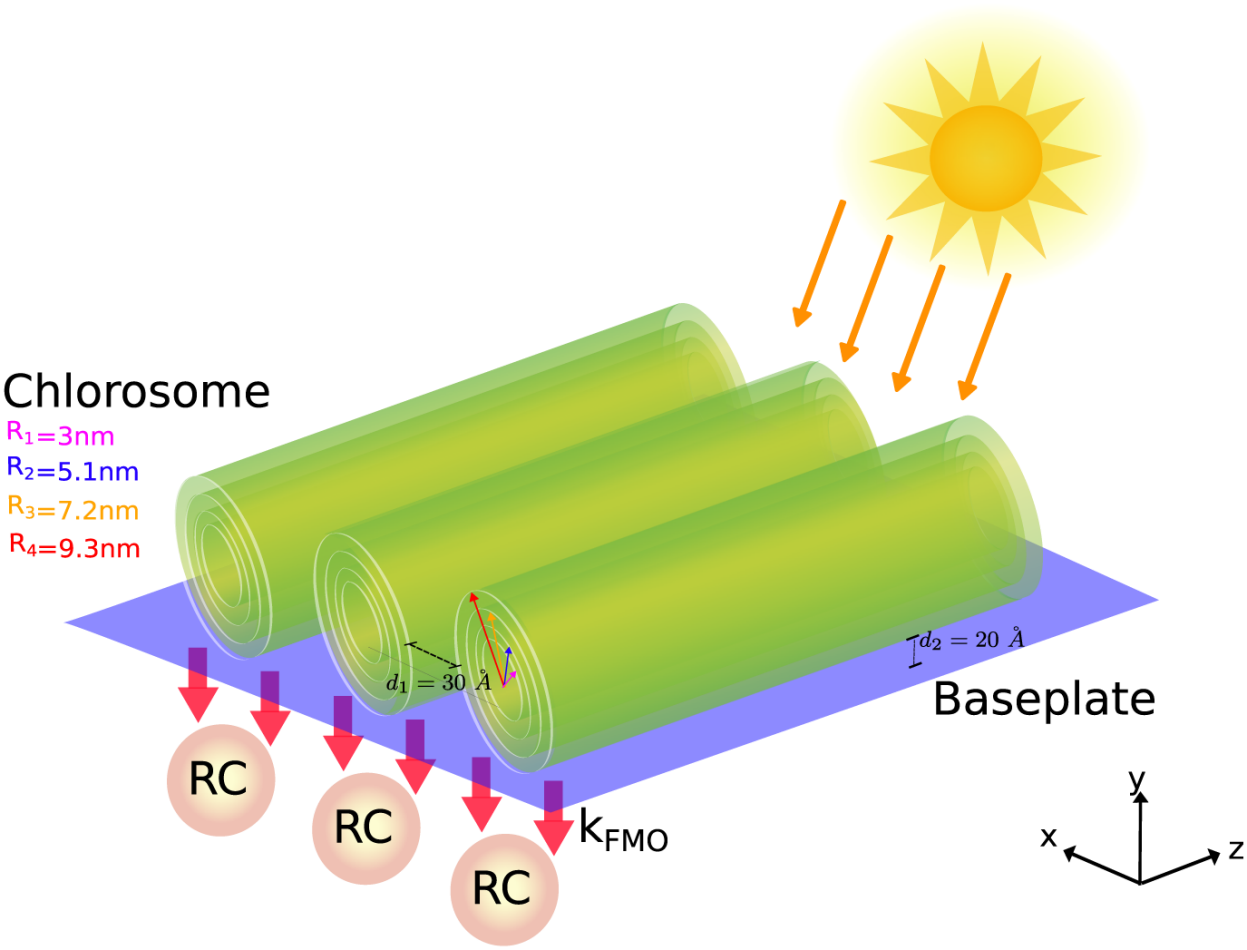
Architecture of GSB light-harvesting unit under natural sunlight. The chlorosome of GSB comprising three adjacent concentric cylinders and the dimeric baseplate (BPL) have been represented. Each aggregate is made of four concentric MT model cylinders with radii of 30, 51, 72, and 93 Å and containing, respectively, 30, 51, 72, and 93 dipoles per ring. The entire chlorosome contains 132840 BChl *c* with a length *L* = 1485.7 Å and the distance between two adjacent concentric cylinders is *d*_1_ = 30 Å. Under the chlorosome a dimeric baseplate with dimesnsions 1147.5 × 3075.3 Å^2^ and comprising 3350 BChl *a* has been represented. The distance between the cylinder and the baseplate is set to *d*_2_ = 20 Å, according to refs 6,16 . Finally the energy transfer from the baseplate to the RCs is mediated by the *k_FMO_* rate, represented by red arrows connecting the baseplate to the RCs.

The basic components of the photosynthetic antenna complexes of anaerobic bacteria have been widely studied both theoretically and experimentally in refs 5,6,29–33 . Due to the symmetric arrangement of BChl molecules, these structures display bright (superradiant) and dark (subradiant) states in their single-excitation manifold.^2,34^ Bright states are characterized by a giant transition dipole moment (much larger than the single-molecule dipole moment), while dark states exhibit a significantly smaller transition dipole moment compared to that of a single molecule.

As already demonstrated in literature,^35^ bright states robust to disorder arise due to the strong coupling between BChl molecules within the aggregates. Within each aggregate (chlorosome and baseplate), excitations are spread coherently due to the small distances between nearest-neighbor BChl molecules (a few angstroms), while between pigment groups at a distance of about a few nanometers, electronic excitation is shared incoherently through multichromophoric Förster resonant energy transfer (MC-FRET).

In this study, we thus conduct a large-scale analysis of sunlight absorption and exciton energy transfer in GSB photosynthetic complexes. Specifically, we examine a model of the GSB chlorosome comprising more than 10^5^ chlorophyll molecules arranged on three adjacent concentric cylinders (the chlorosome) above a two-dimensional dimeric baseplate. In order to understand the relation between shape and functionality, the orientations of BChl dipole moments in the cylindrical structures present in the chlorosome have been modified. The light-harvesting efficiency of the natural geometry has been compared with the light-harvesting efficiency of these mathematical models. Our findings shed new light on how the natural structure of the chlorosome is able to support an efficient energy transfer, even in the presence of thermal noise and static disorder.

## 2 Models: The Geometry of the System

Here we analyze different models of the chlorosome. Together with the natural cylindrical structures present in the chlorosome, we also considered mathematical cylindrical structures where both positions and orientations of the chlorophyll transition dipole moment have been changed with respect to the natural system. Figure 2 shows the three cylindrical models we analyze. The main difference between different models lies in the dipole orientation.

- *Chlorobium tepidum* bchQRU triple mutant (MT). The MT model can be thought as organized into a stack of vertical rings^7,36^ each containing 60 BChl *c* molecules represented by dipoles. In panel A of Figure 2 the alternation between the colors of two consecutive dipoles on the same ring (red and black) represents those dipoles pointing inward (α = +4°) and outward (α = −4°) with respect to the cylinder. See ref 36 for more details.
- Parallel dipoles cylinder (PD). The dipoles on the PD are arranged in circles, similarly to MT type, with their direction parallel to the cylinder main axis, see Figure 2 panel B.
- Random dipoles cylinder (RD). In the RD model, see Figure 2 panel C, the position of the dipoles are the same as in the PD model, while the dipole orientations are randomly chosen from the unit sphere.

**Figure 2:**
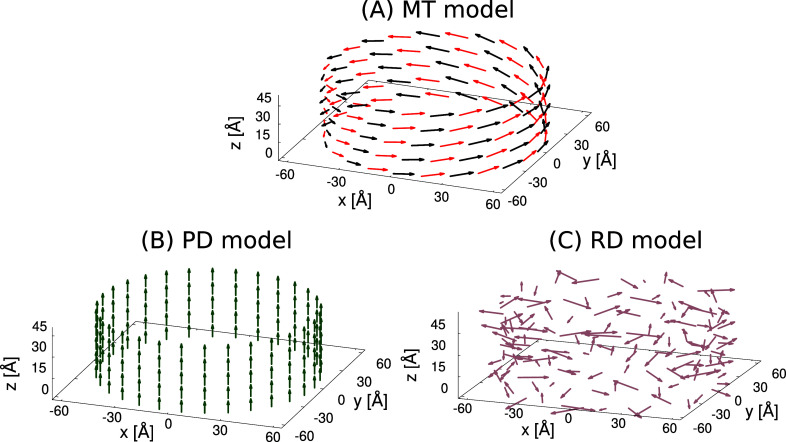
Section of the different cylindrical models. Panel (A) shows the MT model, the structure derived by genetic modification of the natural wild-type model with a radius R = 60 Å. In panels (B–C) PD and RD cylinders made of a stack of rings with the same radius R = 60 Å are presented. For the sake of clarity we show only 30 dipoles per ring instead of 60 as we consider in this paper. For more details about the geometry see refs 36,37 .

The maximum number of molecules we consider in a single cylinder (MT, PD and RD) is *N* = 6000 BChl *c* with a maximum length of 821.7 Å. More information on the geometry of the MT, PD and RD cylinders can be found in refs 36,37 .

The structure of natural GSB light-harvesting systems can vary depending on growing conditions. In general the chlorosome is placed on the top of a two-dimensional dimeric baseplate. The chlorosome does not include only one cylindrical structure as antenna complex, it can contain several multiwall cylindrical structures (usually four concentric cylinders) placed adjacently above the baseplate, see Figure 1. Also BChl molecules aggregated in lateral curved lamellae can be present, depending on the growing conditions.^36–39^

In this manuscript, in order to model the entire light-harvesting unit, we considered a chlorosome composed by three adjacent cylindrical aggregates with four concentric rolls each, containing 132840 BChl *c* molecules and with a length of *L* = 1485.7 Å. Each aggregate is made of four concentric MT model cylinders with radii of 30, 51, 72, and 93 Å and containing, respectively, 30, 51, 72, and 93 dipoles per ring. The distance between two adjacent concentric cylinders is *d*_1_ = 30 Å, according to ref 6.

Below the chlorosome, at a distance *d*_2_ = 20 Å, there is the dimeric baseplate. The baseplate is a two-dimensional aggregate formed by BChl *a* molecules that connects the chlorosome pigments to the RCs through the FMO proteins. It is located on the chlorosome envelope on the surface toward the cytoplasmic membranae^40^ and in nature its dimensions were roughly estimated to be 500 Å × 2000 Å.^6^ Two kind of baseplates are found in literature: monomeric and dimeric.^6,40^ The monomeric baseplate is typical of the FAP (filamentous anoxygenic phototrophs), represented by the *Chloroflexux aurantiacus*, that do not show the FMO complexes,^40^ while the dimeric baseplate is typical of GSB.^6,16^ Even if the microscopic structure of the baseplate has not yet been experimentally verified, a model for the dimeric baseplate lattice found in GSB has already been proposed in refs 16,40 . The lattice has two layers and two different transition dipole moments *μ⃗*_*t*_ and *μ⃗*_*b*_ are used for the top and bottom layers, respectively. Panel A of Figure 3 shows the arrangement of dimers in a portion of the baseplate, distinguishing between blue and red dipoles belonging respectively to the bottom and top layers, while panel B represents the dimeric unit cell comprising *μ⃗*_*t*_ and *μ⃗*_*b*_.

**Figure 3:**
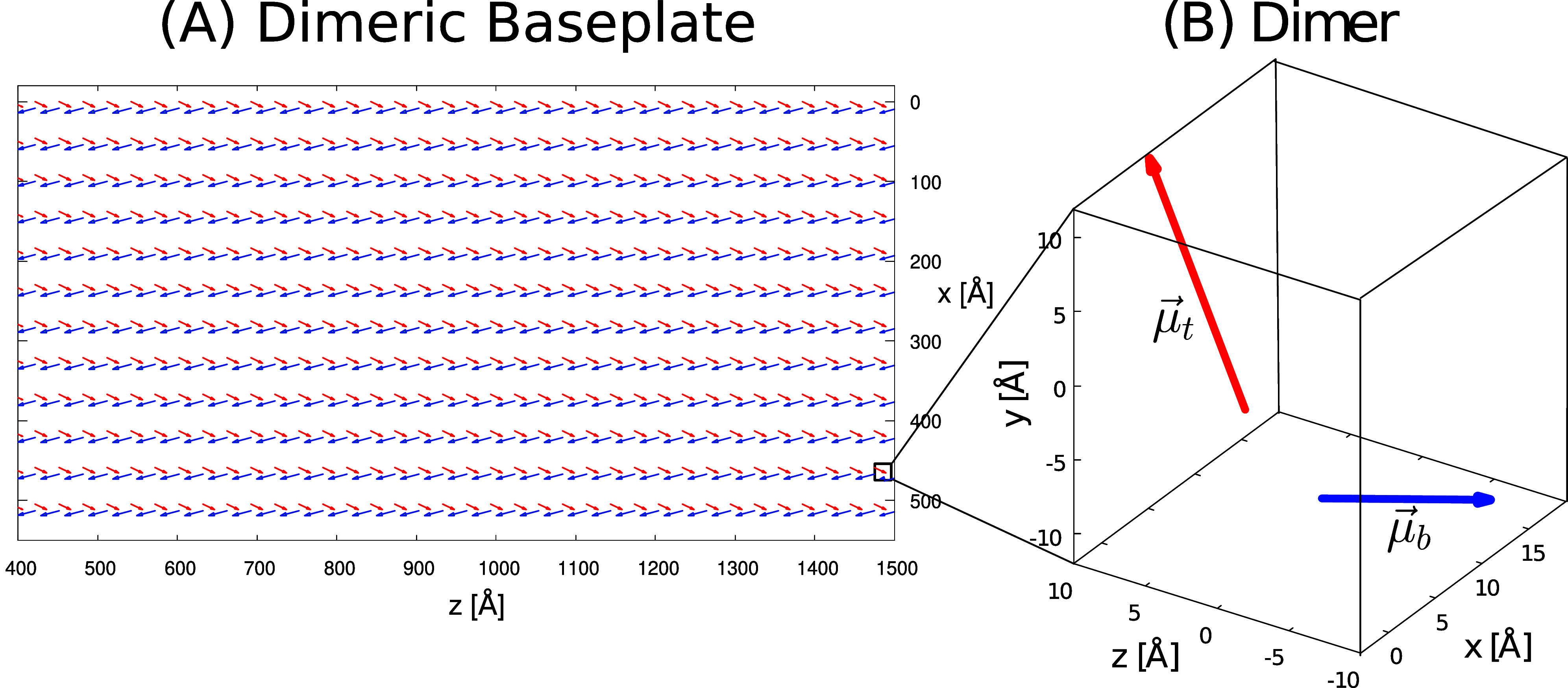
Representation of the dimeric baseplate (BPL) in GSB light-harvesting unit. Panel (A): top view of a portion of the dimeric baseplate, a planar structure in the xz-plane containing BChl *a* molecules. The blue and red arrows represent the TDM associated with each BChl *a* and belonging to the bottom and top layer, respectively. Panel (B): zoomed-in view of the dimeric unit formed by a red arrow (μ*_t_*) and a blue one (μ*_b_*). Dipoles orientations have been found in ref 16, and the corresponding unit vectors are given by *μ̂_t_* = (0.2795, 0.7484, 0.5982) and *μ̂_b_* = (0.2533, 0.1607, −0.9533). The distance between two dipoles in the same dimeric unit cell is set to 12.8 Å, while the distance along consecutive BChl *a* on *x* and *z* axis are 45.9 Å and 30.1 Å respectively. For sake of clarity in panel (B), the dipole length is multiplied by a factor of 16.

After the excitation is absorbed by the BChl molecules in the cylindrical structures or in the baseplate, it is driven to the RCs by the FMO complexes. In GSB light-harvesting systems we have 1 FMO trimer per 50 nm2 ^16^ and 1 RC per 100 nm^2^, since we have one RC every two FMO complexes.

In order to describe the light-harvesting process in the entire photosynthetic complex, a system of incoherent rate equations has been derived, accounting for the interaction of the system with natural sunlight, exciton energy transfer, the interaction with a thermal bath and the presence of dissipation through radiative and nonradiative decay channels.

To this purpose, different levels of approximation have been considered. First, a Lindblad master equation approach has been developed for the populations of the eigenstates of the entire system (single cylinder + baseplate) assuming finite transfer time and finite thermalization time^27,41,42^ (see Section 4). Then, two approximations (see Sections 4.2 and 4.3), decreasing the computational costs, have been developed and compared to the Lindblad approach, finding good agreement.

Before discussing these three approaches, a detailed description of the Hamiltonian of the system is given. For the sake of clarity, in the entire manuscript, the indices *i*, *j* are used to label the site basis (the BChl molecules), while the eigenstates are labeled by *n* and *m*. Finally, three figures of merit have been chosen in order to estimate the efficiency of the energy transfer in the systems here considered: (1) the trapped current to the RCs defined as the number of excitations reaching the RC per unit time under natural sunlight, (2) the internal and (3) external efficiencies, which are the number of excitations reaching the RC divided by the number of incoming photons (external efficiency) or by the number of absorbed photons (internal efficiency).

## 3 The Hamiltonian of the System

Since photosynthetic antennae operate under natural sunlight, which is very dilute, the single-excitation approximation can be used, so that only states containing a single excitation have been considered. Choosing the basis states in the single-excitation manifold, where |*i*⟩ represents a state in which the *i* ^*th*^ molecule is excited while all the others are in the ground state, the systems can be described through a radiative non-Hermitian Hamiltonian (NHH) which accounts for the interaction between the molecules mediated by the electromagnetic field (EMF).^19,20,43,44^ The radiative Hamiltonian reads:

1 $${Ĥ}_{NHH}=\overset{N}{\underset{i=1}{\sum }}e_{0}\left|i\right\rangle \left\langle i\right|+\underset{i\neq j}{\sum }\Delta_{ij}\left|i\right\rangle \left\langle j\right|-\frac{i}{2}\overset{N}{\underset{i,j=1}{\sum }}Q_{ij}\left|i\right\rangle \left\langle j\right|$$

where *e*_0_ = *ℏ*ω_0_ is the excitation energy of single emitter (BChl molecule in our case), with ω_0_ transition frequency. The terms Δ_*ij*_ and *Q*_*ij*_ have a diagonal part given by

2 $$\Delta_{jj}=0,{\;Q}_{jj}=\frac{4}{3}\mu^{2}k_{0}^{3}=\gamma$$

with μ = |*μ⃗*| being the TDM and $k_{0}=\frac{2\pi }{\lambda_{0}}$ , where λ_0_ is the wavelength associated with the molecular transition. The off-diagonal part (*i* ≠ *j*) is given by

3 $$\begin{array}{ll} \Delta_{ij}= & \frac{3\gamma }{4}[\left(-\frac{\cos\left(k_{0}r_{ij}\right)}{\left(k_{0}r_{ij}\right)}+\frac{\sin\left(k_{0}r_{ij}\right)}{{\left(k_{0}r_{ij}\right)}^{2}}+\frac{\cos\left(k_{0}r_{ij}\right)}{{\left(k_{0}r_{ij}\right)}^{3}}\right){\mathrm{\mu ̂}}_{i}\cdot {\mathrm{\mu ̂}}_{j}\\ & -\left(-\frac{\cos\left(k_{0}r_{ij}\right)}{\left(k_{0}r_{ij}\right)}+3\frac{\sin\left(k_{0}r_{ij}\right)}{{\left(k_{0}r_{ij}\right)}^{2}}+3\frac{\cos\left(k_{0}r_{ij}\right)}{{\left(k_{0}r_{ij}\right)}^{3}}\right)\left({\mathrm{\mu ̂}}_{i}\cdot {\mathrm{r̂}}_{ij}\right)\left({\mathrm{\mu ̂}}_{j}\cdot {\mathrm{r̂}}_{ij}\right)] \end{array}$$

4 $$\begin{array}{ll} Q_{ij}= & \frac{3\gamma }{2}[\left(\frac{\sin\left(k_{0}r_{ij}\right)}{\left(k_{0}r_{ij}\right)}+\frac{\cos\left(k_{0}r_{ij}\right)}{{\left(k_{0}r_{ij}\right)}^{2}}-\frac{\sin\left(k_{0}r_{ij}\right)}{{\left(k_{0}r_{ij}\right)}^{3}}\right){\mathrm{\mu ̂}}_{i}\cdot {\mathrm{\mu ̂}}_{j}\\ & -\left(\frac{\sin\left(k_{0}r_{ij}\right)}{\left(k_{0}r_{ij}\right)}+3\frac{\cos\left(k_{0}r_{ij}\right)}{{\left(k_{0}r_{ij}\right)}^{2}}-3\frac{\sin\left(k_{0}r_{ij}\right)}{{\left(k_{0}r_{ij}\right)}^{3}}\right)\left({\mathrm{\mu ̂}}_{i}\cdot {\mathrm{r̂}}_{ij}\right)\left({\mathrm{\mu ̂}}_{j}\cdot {\mathrm{r̂}}_{ij}\right)] \end{array}$$

where *μ̂*_*i*_ ≔ *μ⃗*_*i*_/μ is the unit dipole moment of the *i* ^*th*^ site and *r̂*_*ij*_ ≔ *r⃗*_*ij*_/*r*_*ij*_ is the unit vector joining the *i* ^*th*^ and the *j* ^*th*^ sites. See Table S1 in the Supporting Information for the parameters we used for BChl *a* and *c*.

Diagonalizing the Hamiltonian (eq 1) we obtain the complex eigenvalues $\varepsilon_{n}=E_{n}-i\frac{\Gamma_{n}}{2}$ where Γ_*n*_/*ℏ* is the radiative decay rate of the *n* ^*th*^ eigenstate in s^–1^. In general Γ_*n*_/*ℏ* differs from the radiative decay rate of the single molecule γ/*ℏ*. In particular, when the ratio Γ_*n*_/γ ≫ 1 we will talk about a “superradiant state” (SRS) or bright state, otherwise when Γ_*n*_/γ ≪ 1 the state is called “subradiant” or dark. In other words, an SRS can radiate much faster than a single molecule, while a subradiant one radiates at a rate much slower than the single-molecule radiative decay.

The non-Hermitian part of the radiative Hamiltonian (eq 1) can be treated as a perturbation whenever the decay widths are much smaller than the mean level spacing computed using the real part of the complex eigenvalues, see discussion in Section S5 in the Supporting Information When this criterion, known as *resonance overlap criterion*,^45^ is valid, one can exclusively utilize the Hermitian part of the Hamiltonian. This reduction in complexity accelerates calculations. The Hermitian part of the Hamiltonian (eq 1) is defined as follows:

5 $${Ĥ}_{HH}=\overset{N}{\underset{i=1}{\sum }}e_{0}\left|i\right\rangle \left\langle i\right|+\underset{i\neq j}{\sum }\Delta_{ij}\left|i\right\rangle \left\langle j\right|$$

where Δ_*i*,*j*_ is given in (eq 3). In Section S5 in the Supporting Information a comparison between the radiative decay widths computed with the NHH model for a single cylinder (MT model) and the perturbation theory has been provided. Assuming that we are in the perturbative regime, where the non-Hermitian term *Q*_*ij*_ is much smaller than Δ_*ij*_, the radiative decay widths have been estimated as the expectation value of *Q*_*ij*_ with the eigenstates of the Hermitian Hamiltonian. Our results have demonstrated that for a single cylinder *Q*_*ij*_ can be considered a small perturbation and the same values for the radiative decay widths have been obtained using both the radiative non-Hemrmitian Hamiltonian and the perturbation theory. These foundings have already been proved in ref 37 by some of the authors of this manuscript, where the ratio between all the radiative decay widths Γ_*n*_ and the mean level spacing δ between the energies of the system has been computed for a single cylinder (MT model) and for the entire chlorosome. The results revealed that for the single cylinder the ratio is always smaller than 1, so the perturbative approach is still valid. For the entire chlorosome the maximum value of the ratio, corresponding to the most SRS, is almost 10^2^ and also other eigenstates show a ratio larger than 1, proving that for larger systems the perturbative regime and the HH model fail, while the NHH model is the only way to describe the radiative response of the system.

If the non-Hermitian term *Q*_*ij*_ can be considered a small perturbation and we are in the small-volume limit, when the system size is small compared to the wavelength associated with the optical transition of the molecules (*L* ≪ λ_0_), the radiative decay rate of an eigenstate can be estimated in terms of its dipole strength, computed using only the Hermitian part of the Hamiltonian (HH), see discussion at the end of Section S5 in the Supporting Information Denoting the *n* ^*th*^ eigenstate of the Hermitian part of the Hamiltonian (eq 1) with |*E*_*n*_⟩, we can expand it on the site basis, so that

6 $$\left|E_{n}\right\rangle =\overset{N}{\underset{i=1}{\sum }}C_{n}\left(i\right)\left|i\right\rangle$$

To each basis state |*i*⟩, a dipole moment *μ⃗*_*i*_ is associated, corresponding to the TDM of the *i* ^*th*^ molecule. If *N* is the total number of molecules, then we will express the TDM *D⃗*_*n*_ associated with the *n* ^*th*^ eigenstate as follows:

7 $${\mathrm{D⃗}}_{n}=\overset{N}{\underset{i=1}{\sum }}C_{n}\left(i\right){\mathrm{\mu ̂}}_{i}$$

The dipole strength of the *n* ^*th*^ eigenstate is defined by |*D⃗*_*n*_|^2^ (note that $\overset{N}{\underset{n=1}{\sum }}|{\mathrm{D⃗}}_{n}{|}^{2}=N$ .^46^ Note that when the system size is much smaller than λ_0_ we have |*D⃗*_*n*_|^2^ ≈ Γ_*n*_/γ.

Finally, we note that when resonances do not overlap and the system size is much smaller than λ_0_ (i.e., when *k*_0_ *r*_*ij*_ ≪ 1), the Hermitian part of the radiative Hamiltonian reduces to the standard dipole–dipole Frenkel Hamiltonian (DH):

8 $${Ĥ}_{DH}=\overset{N}{\underset{i=1}{\sum }}e_{0}\left|i\right\rangle \left\langle i\right|+\underset{i\neq j}{\sum }\frac{{\mathrm{\mu ⃗}}_{i}\cdot {\mathrm{\mu ⃗}}_{j}-3\left({\mathrm{\mu ⃗}}_{i}\cdot {\mathrm{r̂}}_{ij}\right)\left({\mathrm{\mu ⃗}}_{j}\cdot {\mathrm{r̂}}_{ij}\right)}{r_{ij}^{3}}\left|i\right\rangle \left\langle j\right|$$

Here we compare the radiative decay widths of the eigenstates of different molecular aggregates computed with the three different Hamiltonian models introduced above:

NHH: non-Hermitian radiative Hamiltonian (eq 1).
HH: Hermitian Hamiltonian (eq 5) valid under the nonoverlapping resonance criterion.
DH: Dipole Hamiltonian (eq 8) valid under the nonoverlapping resonance criterion and when the system size is small compared to the wavelength associated with the optical transition of the molecules.

In Figure 4 the radiative decay widths of the eigenstates obtained by diagonalizing the NHH are compared with the dipole strength computed with the HH and DH models. Note that, both in the main text and in the Supporting Information, all energy values are given in cm^–1^ units, i.e., they are divided by *hc*. Panel A of Figure 4 shows that for the single cylinder, which is about 821.7 Å long, the three models give comparable results, because we are in the small-volume limit, defined as *L* ≪ λ_0_. For the whole chlorosome, see panel B of Figure 4, made of three adjacent concentric cylinders covering an area of 3075.3 × 1147.5 Å^2^ we are neither in the small-volume limit nor in the perturbative one, see discussion in ref 37 and in Section S5 in the Supporting Information Thus, the three Hamiltonians give very different results. For the baseplate, see panel C of Figure 4, of size 2739.1 × 2739.1 Å^2^, the NHH and the HH give very similar results, showing that we are in the perturbative limit with respect to the non-Hermitian interaction. On the other hand, some differences between the DH and the NHH model can be observed. Nevertheless, the discrepancies in the largest dipole strength are just ≈14%, so that we can use the DH as a first approximation.

**Figure 4:**
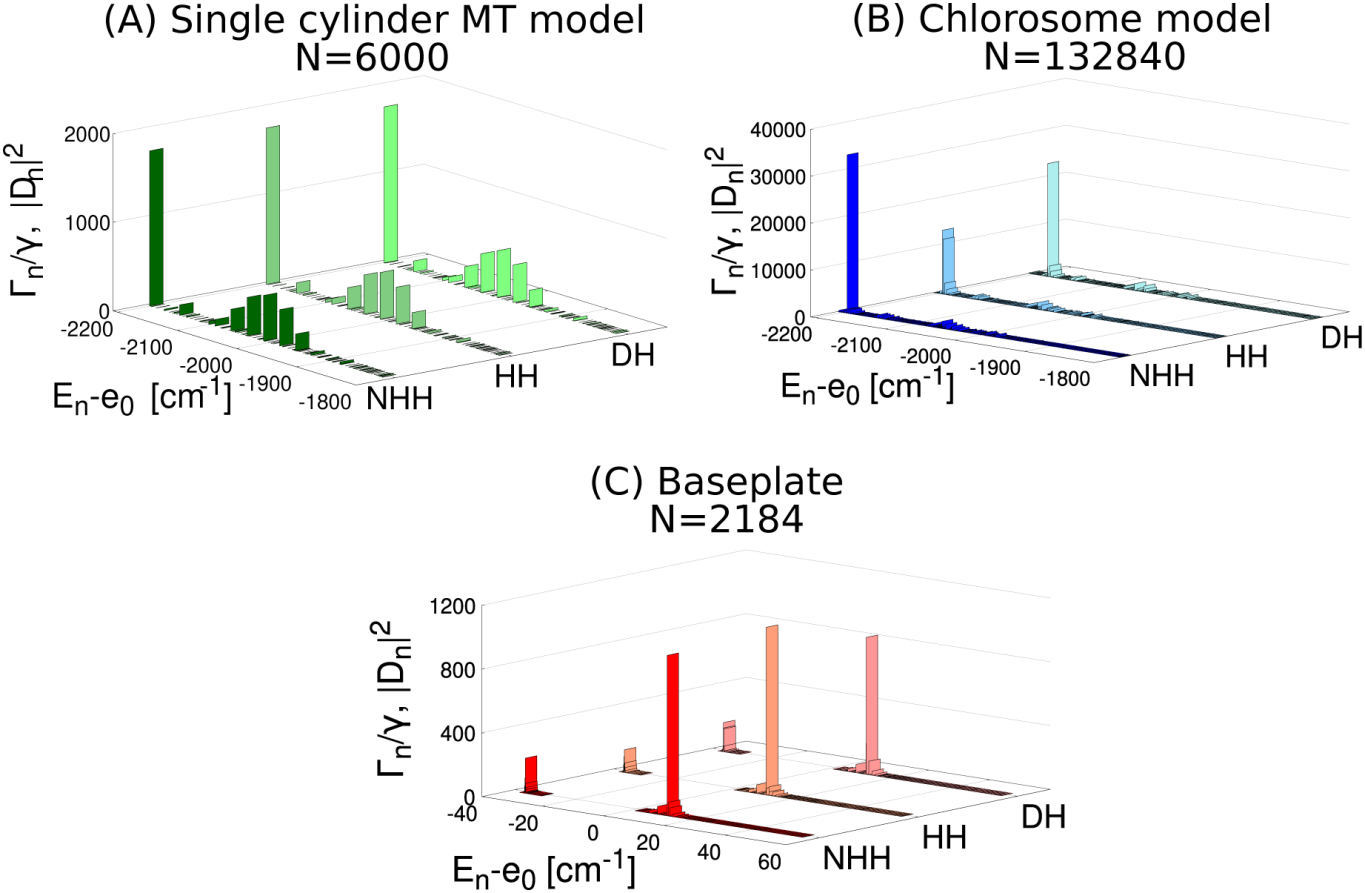
Dipole strength |D_n_|^2^ (DH and HH models) and radiative decay rate Γ_n_/γ (NHH model) in cylindrical aggregates and baseplate. Comparison between the dipole strength (DH and HH models) and radiative decay rate (NHH model) for (A) a single MT cylinder, (B) the chlorosome and (C) the dimeric baseplate. Note that in ref 37, for a single MT cylinder (A) the ratio Γ*_n_*/δ is always less than 1, while for the chlorosome (B) the maximum value of (Γ*_n_*/δ)_max_ is almost 10^2^, proving that the perturbative regime for this aggregate fails and only the NHH model can be used to describe superradiance. The mean level spacing δ has been computed as the ratio between the energy spectral width and the total number of eigenmodes for each complex. For the geometry of the system, refer to Table S3. Panels (A–B) show only the lowest part of the energy spectrum, while panel C represents the entire energy spectrum.

In the following we will use the DH model for the single cylinder and baseplate, while for the whole chlorosome the full NHH Hamiltonian will be used.

## 4 Master Equation

The whole light-harvesting process can be described by the following master equation for the density matrix *ρ̂*_*S*_:^47^

9 $$\frac{d{\mathrm{\rho ̂}}_{S}}{dt}=-\frac{i}{\hbar }\left[{Ĥ}_{S},{\mathrm{\rho ̂}}_{S}\right]+L_{fl}\left[{\mathrm{\rho ̂}}_{S}\right]+L_{Sun}\left[{\mathrm{\rho ̂}}_{S}\right]+L_{T}\left[{\mathrm{\rho ̂}}_{S}\right]$$

that contains the following terms: *Ĥ*_*S*_ is the Hermitian part of the Hamiltonian of the system (according to the aggregate considered we will use either *Ĥ*_*DH*_ or *Ĥ*_*NHH*_, see dicussion above); $L_{fl}$ , $L_{Sun}$ and $L_{T}$ are Lindblad dissipators derived under the Born–Markov and secular approximations.^47^ They describe respectively spontaneous emission $\left(L_{fl}\right)$ and absorption and stimulated emission induced by sunlight $\left(L_{Sun}\right)$ , while $L_{T}$ is the dissipator modeling thermal relaxation and decoherence in the presence of a thermal bath^41,42^ see discussion in Section S6 in the Supporting Information.

The Lindblad dissipators read explicitly:

10a $$L_{fl}\left[{\mathrm{\rho ̂}}_{S}\right]=\underset{ij}{\sum }\frac{Q_{ij}}{\hbar }\left[{â}_{j}{\mathrm{\rho ̂}}_{S}{â}_{i}^{†}-\frac{1}{2}\left\{{â}_{i}^{†}{â}_{j},{\mathrm{\rho ̂}}_{S}\right\}\right]$$

10b $$L_{Sun}\left[{\mathrm{\rho ̂}}_{S}\right]=\underset{ij}{\sum }f_{s}n_{s}\frac{Q_{ij}}{\hbar }\left[{â}_{j}^{†}{\mathrm{\rho ̂}}_{S}{â}_{i}-\frac{1}{2}\left\{{â}_{i}{â}_{j}^{†},{\mathrm{\rho ̂}}_{S}\right\}\right]+\underset{ij}{\sum }f_{s}n_{s}\frac{Q_{ij}}{\hbar }\left[{â}_{j}{\mathrm{\rho ̂}}_{S}{â}_{i}^{†}-\frac{1}{2}\left\{{â}_{i}^{†}{â}_{j},{\mathrm{\rho ̂}}_{S}\right\}\right]$$

where the sums over *i*, *j* run over all the system sites, *â*_*i*_ = |0⟩⟨*i*| (|0⟩ is the ground state with no excitation, while ⟨*i*| can be a cylinder or baseplate site), *Q*_*ij*_ is given in (eq 4) and in the small-volume limit *Q*_*ij*_ ≈ γ*μ̂*_*i*_ · *μ̂*_*j*_.

Note that the absorption of sunlight photons is non-Markovian and leads to coherent oscillations, i.e., Fano coherence, even if the excitation is incoherent. The sunlight-induced coherence is particularly relevant as the dipole coupling in (eq 4) is collective. As demonstrated in an early calculation, the sunlight-induced coherence may not affect light-harvesting efficiency, as dephasing is typically faster than energy transfer. Then, the Markov approximation assumed in the quantum master equation can be justified.^48^

Finally $L_{T}$ is

11 $$L_{T}\left[{\mathrm{\rho ̂}}_{S}\right]=\underset{\omega }{\sum }\gamma^{\left(p\right)}\left(\omega \right)\underset{i}{\sum }\left[{Â}_{i}\left(\omega \right){\mathrm{\rho ̂}}_{S}{Â}_{i}^{†}\left(\omega \right)-\frac{1}{2}\left\{{Â}_{i}^{†}\left(\omega \right){Â}_{i}\left(\omega \right),{\mathrm{\rho ̂}}_{S}\right\}\right]$$

where

12 $$\gamma^{\left(p\right)}\left(\omega \right)=2\pi \left[J\left(\omega \right)\left(1+n_{BE}\left(\omega \right)\right)+J\left(-\omega \right)n_{BE}\left(-\omega \right)\right]$$

are the thermal rates, depending of the spectral density *J*(ω) and on the Bose distribution $n_{BE}\left(\omega \right)=(e^{\hbar \omega /k_{B}T}-1{)}^{-1}$ of the phonons at room temperature (*T* = 300 K) and

13 $${Â}_{i}\left(\omega \right)=\underset{E_{m}-E_{n}=\hbar \omega }{\sum }C_{m}^{*}\left(i\right)C_{n}\left(i\right)\left|E_{n}\right\rangle \left\langle E_{m}\right|$$

More details about the Lindblad dissipators can be found in refs 41,42 .

Equation 9, in its Lindblad form, is derived under the secular approximation. Moreover, it can be largely simplified under a well-motivated assumption: the excitons are transferred incoherently between the cylinder eigenstates and baseplate eigenstates; this is consistent with the approach of some recent works^41,42,49,50^ where a similar model to describe exciton dynamics in GSB antenna complexes has been already employed. Under these assumptions, the coupling with the thermal bath and natural sunlight are well approximated by rate equations for the populations of the cylinder eigenstates (*P*_*n∈C*_) and the populations of the baseplate eigenstates (*P*_*n∈B*_), as we show in the following.

### 4.1 Rate Equations for the Light-Harvesting Process: Finite Transfer Time and Finite Thermalization Time

The following set of full rate equations for the whole light-harvesting process have been derived starting from the master eq 9 (see also Section S6 of the Supporting Information). They read:

14a $$\begin{array}{ll} \frac{dP_{0}\left(t\right)}{dt}= & -\underset{n}{\sum }R_{n}P_{0}\left(t\right)+\underset{n}{\sum }\left(R_{n}+\Gamma_{n}/\hbar +\kappa_{NR}\right)P_{n}\left(t\right)\\ & +\underset{n\in B}{\sum }\kappa P_{n}\left(t\right) \end{array}$$

14b $$\begin{array}{ll} \frac{dP_{n\in C}\left(t\right)}{dt}= & R_{n}P_{0}\left(t\right)-\left(R_{n}+\Gamma_{n}/\hbar +\kappa_{NR}\right)P_{n}\left(t\right)\\ & +\underset{m\in C}{\sum }\left(T_{n,m}P_{m}\left(t\right)-T_{m,n}P_{n}\left(t\right)\right)\\ & +\underset{m\in B}{\sum }\left(K_{n,m}P_{m}\left(t\right)-K_{m,n}P_{n}\left(t\right)\right) \end{array}$$

14c $$\begin{array}{ll} \frac{dP_{n\in B}\left(t\right)}{dt}= & R_{n}P_{0}\left(t\right)-\left(R_{n}+\Gamma_{n}/\hbar +\kappa_{NR}+\kappa \right)P_{n}\left(t\right)\\ & +\underset{m\in B}{\sum }\left(T_{n,m}P_{m}\left(t\right)-T_{m,n}P_{n}\left(t\right)\right)\\ & +\underset{m\in C}{\sum }\left(K_{n,m}P_{m}\left(t\right)-K_{m,n}P_{n}\left(t\right)\right) \end{array}$$

where the summations ∑*_n∈C_* include all the cylinder eigenstates, while ∑*_n∈B_* include all the baseplate eigenstates and ∑_*n*_ include all the eigenstates (cylinder and baseplate).

The terms in eq 15(a–c) are explained in detail in the following.

#### 4.1.1 Radiative Decay

The radiative decay Γ_*n*_/*ℏ* is given by the imaginary part of the NHH. When the DH is valid, it can be computed from the dipole strengths of the eigenstates as follows:

15 $$\frac{\Gamma_{n}}{\hbar }=\frac{4}{3}\frac{\mu^{2}D_{n}^{2}\omega_{n}^{3}}{\hbar c^{3}}$$

where *D*_*n*_ is defined in (eq 7) and ω_*n*_ = *E*_*n*_/*ℏ* is the eigenstate transition frequency.

#### 4.1.2 Absorption and Stimulated Emission Induced by Sunlight Radiation

Sunlight induces absorption and stimulated emission to each eigenstate |*E*_*n*_⟩ with rates given by

16 $$R_{n}=f_{S}n_{S}\left(\omega_{n}\right)\frac{\Gamma_{n}}{\hbar }$$

where

17 $$n_{S}\left(\omega_{n}\right)=\frac{1}{e^{\hbar \omega_{n}/(k_{B}T_{S})}-1}$$

is the Bose occupation of photons at the blackbody temperature of the Sun, *T*_*S*_ = 5800 K, while the factor

18 $$f_{S}=\frac{\pi r_{S}^{2}}{4\pi R_{ES}^{2}}=5.4\times 10^{-6}$$

models how the absorption (and stimulated emission) rate is reduced by the Sun–Earth distance.^41^ Specifically, *f*_*S*_ is the fraction of the solid angle of the Sun as seen from the Earth, with *r*_*S*_ being the radius of the Sun and *R*_*ES*_ the Sun–Earth distance. See Section S1 in the Supporting Information for a more detailed discussion about the validity of the approximation of the solar spectrum with the blackbody radiation theory and Section S2.2 in the Supporting Information for a comparison between different approaches used to compute the absorption rates of BChl molecules. Note that the solar spectrum is not exactly described by blackbody radiation, as the photon experiences multiple scatterings upon arriving on the Earth. However, the sunlight spectrum is sufficiently broad compared to the absorption of light-harvesting complexes and our approximation holds.^48^

#### 4.1.3 Nonradiative Decay

In addition to the radiative decay, we include nonradiative recombination processes on each *n* eigenstate by adding a nonradiative rate κ_*NR*_ = 1 ns^–1^ for each eigenstate.^27^

#### 4.1.4 Trapping to RC

We also consider an additional decay channel due to excitation transfer from the baseplate to the reaction centers (RCs) through the FMO trimers. We model this by adding a decay rate κ on all the baseplate eigenstates. Specifically, the excitation is lost through the FMOs to the RCs with a rate *k*_*FMO*_ ∼ 0.023–0.044 ps^–1^.^26^ *k*_*FMO*_ is the reciprocal of the time required for an excitation to be lost in the RC through the FMO, and such time is the sum of four contributions,

19 $$k_{FMO}^{-1}=\tau_{b}+\tau_{t}+\tau_{e}+\tau_{cs}$$

that represent explicitly:

- the transfer time τ_*b*_ *from the baseplate to the FMO*, which we estimate as the inverse of the FRET rate
20 $$\tau_{b}=\frac{\hbar^{2}\Gamma_{\phi }}{2J^{2}}$$
  between the two molecules closest to each other, one in the baseplate and one in the FMO complex; here, Γ*_ϕ_* = 1000 cm^–1^, where Γ*_ϕ_*/*ℏ* is the dephasing rate, estimated from the cylinder-baseplate transition rate, as explained in Section 4.2; *J* ≈ μ^2^/*r* ^3^ is the dipole coupling between two BChl molecules (one belonging to the baseplate and the other one to the FMO), where μ ≈ 6 D is the transition dipole of a single BChl molecule and *r* = 20 Å is the distance; therefore we have *J* ≈ 23 cm^–1^, from which τ_*b*_ ≈ 5 ps;
- the time for an excitation to go *through the FMO* from edge to edge has been estimated in ref 51, and it is τ_*t*_ ≈ 3 ps, in good agreement with the experimental data found in ref 26, that report an energy relaxation within the FMO complex, which lies between 0.1 and 20 ps;
- the exit time τ_*e*_ *from FMO to RC*, which is ∼17 ps;^26^
- the *charge-separation* time τ_*cs*_ at which the excitation is irreversibly lost in the RC is τ_*cs*_ ≈ 1 ps.^2,26^

The sum of these three contributions gives the typical range $22.5\;\mathrm{ps}<k_{FMO}^{-1}<43\;\mathrm{ps}$ , in agreement with the estimation given above.

Finally the trapping rate to the RC κ can be estimated as follows. We have a molecular density of 1 molecule per 6.9 nm^2^ in the baseplate and a density of 1 FMO trimer per 50 nm^2^.^16^ Since we have one RC every two FMO trimers^23,39,52,53^ we have a density of 1 RC per 100 nm^2^. Therefore, we have a ratio *N*_*FMO*_/*N*_*BPL*_ = 0.138 between the number of FMO trimers and the number of baseplate molecules. So, we have modeled the FMO-RC trapping with a constant decay rate κ = 0.138 *k*_*FMO*_ from all the baseplate molecules (see eq 15(a–c)).

#### 4.1.5 Thermal Relaxation and Energy Transfer

Finally, we include thermal relaxation rates *T*_*n*,*m*_ between each pair of eigenstates within the cylinder and the baseplate, and Förster energy transfer rates *K*_*n*,*m*_ between the cylinder and the baseplate. More details on this are given below.

The transfer rates *T*_*m*,*n*_ model thermal relaxation inside the aggregate, see Section S6 in Supporting Information for the derivation. They are detailed-balanced, namely

21 $$T_{m,n}=\frac{2\pi \Lambda_{mn}J\left[\left(E_{m}-E_{n}\right)/\hbar \right]}{1-e^{-(E_{m}-E_{n})/(k_{B}T)}}$$

where Λ_*mn*_ = ∑_*i*_|*C*_*m*_(*i*)|^2^|*C*_*n*_(*i*)|^2^, the phonon temperature is *T* = 300 K and the spectral density is *J*(ω) = κ_*vib*_ω · κ_*vib*_ = 0.3 ensures thermal relaxation in a few-picoseconds time scale.^41^ On the other hand, the transfer rates between eigenstates of different aggregates are the incoherent Förster rates

22 $$K_{m,n}=\left\{ \begin{array}{ll} \frac{2\Omega_{m,n}^{2}\Gamma_{\phi }}{\hbar \left[\Gamma_{\phi }^{2}+{\left(E_{m}-E_{n}\right)}^{2}\right]} & E_{n}\geq E_{m}\\ \frac{2\Omega_{m,n}^{2}\Gamma_{\phi }}{\hbar \left[\Gamma_{\phi }^{2}+{\left(E_{m}-E_{n}\right)}^{2}\right]}e^{-(E_{m}-E_{n})/(k_{B}T)} & E_{n}<E_{m} \end{array}\right.$$

where Ω_*m*,*n*_ is the dipole–dipole coupling (matrix element) between the eigenstates of different aggregates computed by using (eq 8), while Γ*_ϕ_* = 1000 cm^–1^, where Γ*_ϕ_*/*ℏ* is the dephasing rate for every *m*–*n* transition and *E*_*n*_ is the transition energy of the *n*th eigenstate. Note that the “energy-upwards” rates in eq 25 are multiplied by an exponential factor that ensures the detailed balance.^28^ The parameter Γ*_ϕ_* has been tuned to match the transfer rates between the MT cylinder and the baseplate (tens of picoseconds^16,54,55^ ) and it corresponds to the sum of the HWHM of the emission spectrum of the donor and the absorption spectrum of the acceptor. See Section S7 for a detailed discussion about FRET and Section S8 in the Supporting Information for a clear validation of the parameters employed in this work, specifically regarding the choice of Γ*_ϕ_*, the intramolecular coupling strength and the intrinsic radiative decay rate of the Bchl molecules.

The rate eq 15(a–c) is linear and they can be solved at the steady state or as a function of time by numerical diagonalization, with a computational cost that increases as *N* ^3^, where *N* is the total number of molecules in the cylinder + baseplate. A scheme of eq 15(a–c) is shown in Figure 5.

**Figure 5:**
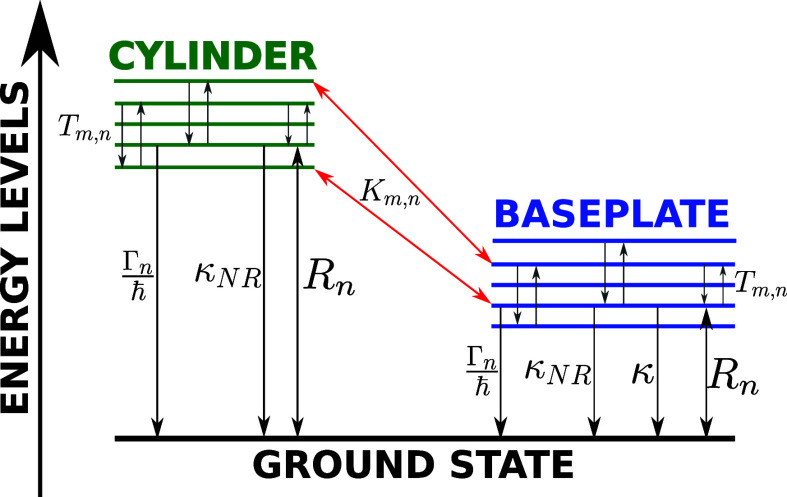
Rate equation scheme. Scheme of the rate equations, eq 15(a–c). Here, Γ*_n_*/*ℏ* is the radiative recombination rate, κ*_NR_* is the nonradiative recombination rate (equal for all levels), $R_{n}=f_{S}n_{S}\left(\omega_{n}\right)\frac{\Gamma_{n}}{\hbar }$ are the sunlight absorption and stimulated emission rates, *T*_*m*,*n*_ are the thermalization rates within each aggregate, see eq 24, *K*_*m*,*n*_ are the transfer rates between eigenstates of each aggregates, see eq 25, and κ is the trapping rate from the baseplate, through the FMOs, to the RC.

From the steady-state solution of eq 15(a–c) we obtain the current trapped in the RCs,

23 $$I_{RCs}=\kappa \underset{n\in B}{\sum }P_{n}^{SS}$$

where $P_{n}^{SS}$ are steady-state populations of the baseplate eigenstates, and we also compute the internal efficiency,^28^

24 $$\eta_{in}=\frac{\kappa \underset{n\in B}{\sum }P_{n}^{SS}}{\underset{n}{\sum }R_{n}P_{0}^{SS}}$$

where $P_{0}^{SS}$ is the steady-state population of the ground state and the sum in the denominator runs over all the cylinder and baseplate states.

Here we introduce another figure of merit, the external efficiency:

25 $$\eta_{ext}=\frac{I_{RCs}}{I_{Sun}\times A_{BPL}}=\eta_{abs}\times \eta_{in}$$

where *I*_*Sun*_ is the photon flux coming from sunlight impinging the baseplate surface, *I*_*RCs*_ is the total current trapped in the RCs, η_*abs*_ and η_*in*_ are the absorption and internal efficiencies, respectively. An analytical expression for η_*in*_ has been given in eq 27, while the absorption efficiency η_*abs*_, given by (eq S11) in the Supporting Information, is the ratio between the power absorbed by the system and the solar irradiance. Section S3 in the Supporting Information offers a deeper study and detailed calculations of the absorption in the entire light-harvesting apparatus of GSB comprising the chlorosome and the baseplate.

### 4.2 Thermal Equilibrium within Each Aggregate

To simplify the rate eq 15(a–c), here we follow a widely used approach, presented e.g., in refs 56,57 and in ref 2. In this approach, thermal equilibrium is assumed inside each aggregate (a cylinder, or the baseplate). The full rate equations shown in eq 15(a–c) is the most accurate model, however, due to the large size of our systems, a less demanding approach, the partially thermalized model, is derived. This approach is valid when thermal relaxation within each aggregate is the fastest time scales. Under this approximation the energy transfer process between aggregates is well captured by the MC-FRET theory, which is valid when the transfer rate between aggregates is much smaller than the thermalization rate within each aggregate. Note that light-harvesting systems are usually disordered and strongly coupled to protein environments, so the thermalization process may not lead to the Boltzmann distribution of the system Hamiltonian.^58^ Instead, the light-harvesting system relaxes to noncanonical distribution due to the system-bath correlation and exhibit bath-induced coherence, which can affect fluorescence emission and Förster energy transfer. The Master Equation in eq 9 assumes the factorization of the system-bath density matrix and thus implies the system Boltzmann distribution. Possible corrections due to the system-bath correlation can be considered in the future.

Under the assumption of thermalization in each aggregate, the transfer rate from a donor aggregate “D” to an acceptor aggregate “A” is given by the generalized multichromophoric Förster resonance energy transfer rate (MC-FRET)

26 $$K_{D,A}=\underset{m\in D}{\sum }\underset{n\in A}{\sum }\frac{e^{-E_{m}/(k_{B}T)}}{\underset{l\in D}{\sum }e^{-E_{l}/(k_{B}T)}}K_{n,m}$$

where *K*_*n*,*m*_ is the transfer rate from the donor eigenstate *m* to the acceptor eigenstate *n*, see eq 25. Section S7 in the Supporting Information gives a more detailed explanation of the MC-FRET. Similarly, we assume thermal equilibrium within each aggregate to compute the decay rates, as explained in the following. We obtain the following 3-level rate equations,

27a $$\begin{array}{ll} \frac{dP_{0}\left(t\right)}{dt}= & -\left(R_{C}+R_{B}\right)P_{0}\left(t\right)+\left(\langle R\rangle_{C}+\langle \gamma \rangle_{C}\right)P_{C}\left(t\right)+\left(\langle R\rangle_{B}+\langle \gamma \rangle_{B}\right)P_{B}\left(t\right)+\kappa P_{B}\left(t\right)\\ & +\kappa_{NR}\left[P_{C}\left(t\right)+P_{B}\left(t\right)\right] \end{array}$$

27b $$\frac{dP_{C}\left(t\right)}{dt}=R_{C}P_{0}\left(t\right)-\left(\langle R\rangle_{C}+\langle \gamma \rangle_{C}\right)P_{C}\left(t\right)+K_{B,C}P_{B}\left(t\right)-K_{C,B}P_{C}\left(t\right)-\kappa_{NR}P_{C}\left(t\right)$$

27c $$\begin{array}{ll} \frac{dP_{B}\left(t\right)}{dt}= & R_{B}P_{0}\left(t\right)-\left(\langle R\rangle_{B}+\langle \gamma \rangle_{B}\right)P_{B}\left(t\right)+K_{C,B}P_{C}\left(t\right)-K_{B,C}P_{B}\left(t\right)-\kappa P_{B}\left(t\right)\\ & -\kappa_{NR}P_{B}\left(t\right) \end{array}$$

where *P*_0_(*t*), *P*_*C*_(*t*) and *P*_*B*_(*t*) are the populations of the ground state, the cylinder and the baseplate, respectively. *R*_*C*_ = ∑*_n∈C_R*_*n*_ and *R*_*B*_ = ∑*_n∈B_R*_*n*_ are the total absorption rates of the cylinder and the baseplate, respectively. (⟨*R*⟩_*C*_ + ⟨γ⟩_*C*_) and (⟨*R*⟩_*B*_ + ⟨γ⟩_*B*_) are the thermal-averaged emission rates, given by

28 $$\langle R\rangle_{C,B}+\langle \gamma \rangle_{C,B}=\underset{n\in C,B}{\sum }\left(R_{n}+\Gamma_{n}/\hbar \right)p_{n}$$

of the cylinder (*C*) and the baseplate (*B*) with the Boltzmann weights within each aggregate

29 $$p_{n}=\frac{e^{-E_{n}/(k_{B}T)}}{\underset{m\in C,B}{\sum }e-E_{m}/(k_{B}T)}$$

*K*_*C*,*B*_, *K*_*B*,*B*_ are donor–acceptor rates computed from eq 29 using the Lorentzian lineshapes as in eq 25.

The collective Förster energy transfer between two aggregates often exhibits simple scaling laws as a function of distance, site and orientation. Using the generalized MC-FRET, we analyzed the scaling laws for interwire energy transfer.^59^ This approach can be applied to other geometries and was recently adopted for the chlorosome lamellae.^60,61^ The reported results on energy transfer can be understood in this framework.

We numerically solve eq 30(a–c) at the steady-state to obtain the number of excitations trapped in the RCs per unit time,

30 $$I_{RCs}=\kappa P_{B}^{SS}$$

We also compute the internal efficiency as the ratio between the excitations trapped in the RC per unit time and the excitations absorbed per unit time,^28^

31 $$\eta_{in}=\frac{\kappa P_{B}^{SS}}{\left(R_{C}+R_{B}\right)P_{0}^{SS}}$$

### 4.3 Thermal Equilibrium among All the Aggregates

If the transfer rate between the aggregates is very fast, we can make a further approximation and assume that all the aggregates are at thermal equilibrium between one another. In such case, let us recall the rate equation for the population of the ground state, eq 15

32 $$\begin{array}{ll} \frac{dP_{0}\left(t\right)}{dt}= & -\underset{n}{\sum }R_{n}P_{0}\left(t\right)+\underset{n}{\sum }R_{n}P_{n}\left(t\right)\\ & +\underset{n}{\sum }\frac{\Gamma_{n}}{\hbar }P_{n}\left(t\right)+\underset{n}{\sum }\kappa_{NR}P_{n}\left(t\right)+\underset{n\in B}{\sum }\kappa P_{n}\left(t\right) \end{array}$$

where *P*_0_ is the population of the ground state and *P*_*n*_ is the population of the *n*-th excitonic eigenstate.

Then, let us assume that the whole single-excitation subspace is at thermal equilibrium with a temperature *T* = 300 K. This approximation is reasonable in the case where the thermalization process among all the aggregates (between cylinders, or between cylinders and the baseplate) happens faster than the superradiant radiative decay of the cylinders. In this case, we define the population in the excited subspace of the whole system at time *t* as

33 $$P_{e}\left(t\right)=\overset{N}{\underset{n=1}{\sum }}P_{n}\left(t\right)$$

so that the trace preservation condition can be written as

34 $$P_{0}\left(t\right)+P_{e}\left(t\right)=1$$

and, crucially, we assume that

35 $$P_{n}\left(t\right)=P_{e}\left(t\right)p_{n}\;\mathrm{with}\;p_{n}=\frac{e^{-E_{n}/(k_{B}T)}}{\underset{m}{\sum }e^{-E_{m}/(k_{B}T)}}$$

By substituting (eq 40) into (eq 37) we have

36 $$\begin{array}{ll} \frac{dP_{0}\left(t\right)}{dt}= & -\left(\underset{n}{\sum }R_{n}\right)P_{0}\left(t\right)+\left(\underset{n}{\sum }R_{n}p_{n}\right)P_{e}\left(t\right)\\ & +\left(\underset{n}{\sum }\frac{\Gamma_{n}}{\hbar }p_{n}\right)P_{e}\left(t\right)+\kappa_{NR}\left(\underset{n}{\sum }p_{n}\right)P_{e}\left(t\right)\\ & +\kappa \left(\underset{n\in B}{\sum }p_{n}\right)P_{e}\left(t\right) \end{array}$$

Equation 41 can be expressed in terms of the thermal averages of the parameters,

37 $$\left\langle R\right\rangle =\underset{n}{\sum }R_{n}p_{n},\;\left\langle \gamma \right\rangle =\underset{n}{\sum }\frac{\Gamma_{n}}{\hbar }p_{n}$$

noting that ∑_*n*_ *p*_*n*_ = 1, defining the fraction of the excited population on the baseplate *p*_*B*_ = ∑*_n∈B_p*_*n*_, and by defining the total absorption rate as *R*_*TOT*_ = ∑_*n*_ *R*_*n*_ we get

38 $$\frac{dP_{0}\left(t\right)}{dt}=-R_{TOT}P_{0}\left(t\right)+\left(\left\langle R\right\rangle +\left\langle \gamma \right\rangle +\kappa_{NR}+\kappa p_{B}\right)P_{e}\left(t\right)$$

The current trapped at the steady state is obtained by using eq 39:

39 $$I_{RCs}=\kappa p_{B}P_{e}^{SS}=\frac{\kappa p_{B}R_{TOT}}{R_{TOT}+\left\langle R\right\rangle +\left\langle \gamma \right\rangle +\kappa_{NR}+\kappa p_{B}}$$

while the internal efficiency is

40 $$\eta_{in}=\frac{\kappa p_{B}P_{e}^{SS}}{R_{TOT}P_{0}^{SS}}=\frac{\kappa p_{B}}{\left\langle R\right\rangle +\left\langle \gamma \right\rangle +\kappa_{NR}+\kappa p_{B}}$$

Here, $P_{e}^{SS}$ represents the total excitonic population in the system at the steady state, under natural sunlight illumination, assuming thermal equilibrium in the whole system and accounting for radiative and nonradiative recombination. This method only requires the knowledge of the radiative and nonradiative decay rates of the eigenstates and of their energies.

Note that, since the baseplate is well gapped below the cylinder, we have *p*_*B*_ ≈ 1. Moreover, for κ_*NR*_ = 1 ns^–1^, we also typically have ⟨*R*⟩ + ⟨γ⟩ ≪ κ_*NR*_. Therefore, the internal efficiency has the approximate expression:

41 $$\eta_{in}\approx \frac{\kappa }{\kappa_{NR}+\kappa }$$

## 5 Results and Discussions

### 5.1 Exciton Energy Transfer in GSB Light-Harvesting Systems

In this section the main results of this study are presented comparing the natural structure with the light-harvesting systems obtained by modifying the orientation of the dipoles in the cylindrical aggregates.

First we consider a single-wall nanotube coupled to a baseplate where the excitation can be trapped with a trapping rate *k*_*FMO*_. For the single-wall nanotubes, we consider three different types of cylindrical aggregates (MT, PD and RD, see Section 2). All single-wall cylinders are composed of 6000 BChl molecules and they are 821.7 Å long. The baseplate coupled to the cylindrical structures is composed of 2184 BChl molecules and has an area of 550.8 × 2739.1 Å^2^. In addition to the single-wall nanotubes, we also consider the full model of the GSB photosynthetic antenna (chlorosome) composed of 132840 BChl molecules distributed among three MT adjacent concentric cylinders (four wall each), see Section 2. Each concentric cylinder includes 44280 BChl molecules, and the baseplate for the whole chlorosome is composed of 3350 molecules and with an area of 1145.7 × 3075.3 Å^2^, see Table S3 in Section S4 of the Supporting Information where a more detailed explanation of the geometrical features of the cylindrical aggregates and baseplate is given.

For each light-harvesting complex we computed the trapped current (Figures 6 and 7 panel A) and the internal efficiency (Figures 6 and 7 panel B). The external efficiency has been computed for the entire chlorosome coupled to a baseplate (Figure 7 panel C), and for all the other single-cylinder models, see Section S3 of the Supporting Information.

**Figure 6:**
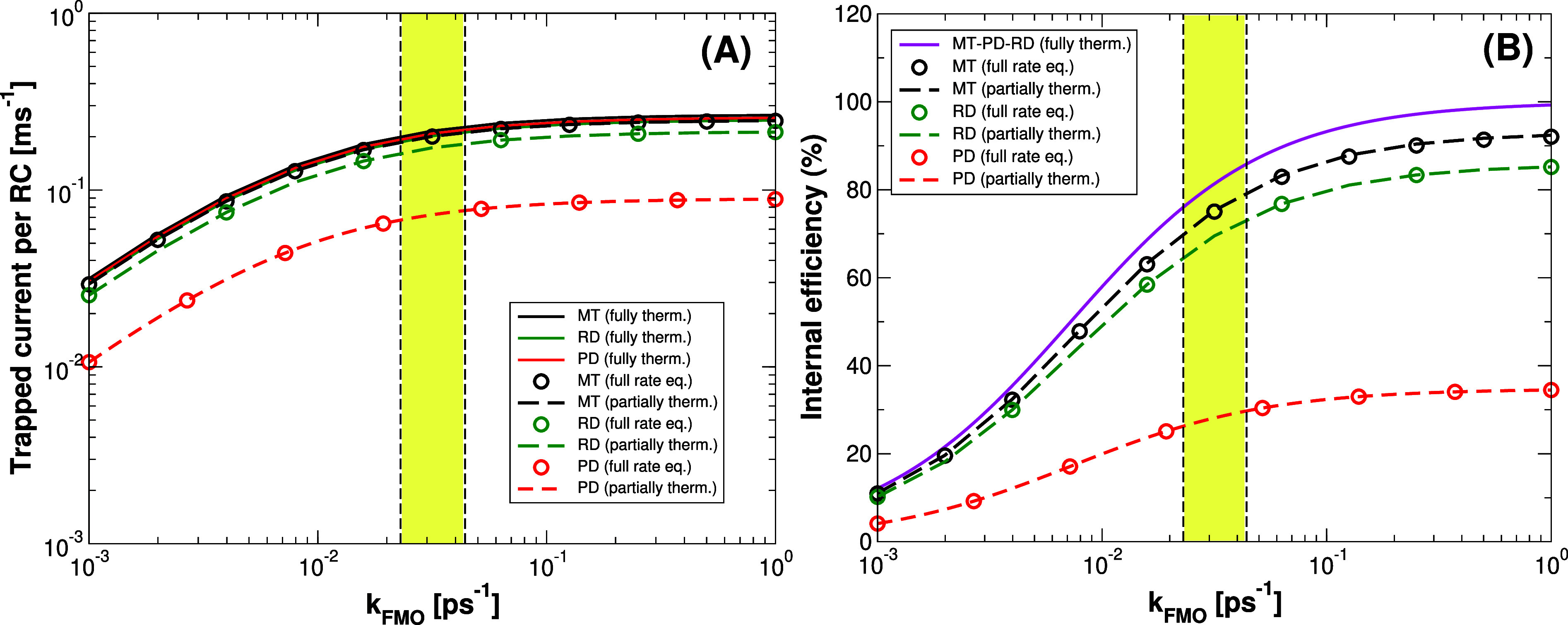
Trapped current and internal efficiency vs transfer rate k_FMO_from baseplate to RCs for single-cylinder models. Trapped current per RC (panel A) and internal efficiency (panel B) for the single-cylinder models (MT, PD, RD coupled to the dimeric baseplate) comprising 6000 BChl *c* in the cylinder and 2184 BChl *a* in the baseplate. More details about the dimensions of the systems can be found in Table S3 of the Supporting Information. Black, green and red dashed lines have been obtained assuming thermalization in each aggregate and solving eq 30(a–c) in MT, RD and PD models, respectively. Open circles represents the results obtained by solving the full rate equations given in eq 15(a–c). Continuous lines represent the trapped current and internal efficiency assuming thermalization among all aggregates, obtained from eq 37. According to eq 46 the internal efficiency has a universal expression for all the models. The yellow box between the two vertical dashed lines represents the regime in which FMO complexes typically work (0.023 ps^–1^ < *k_FMO_* < 0.044 ps^–1^). Trapped current and internal efficiency for the RD model have been computed averaging over 10 realizations of random dipole orientations.

**Figure 7:**
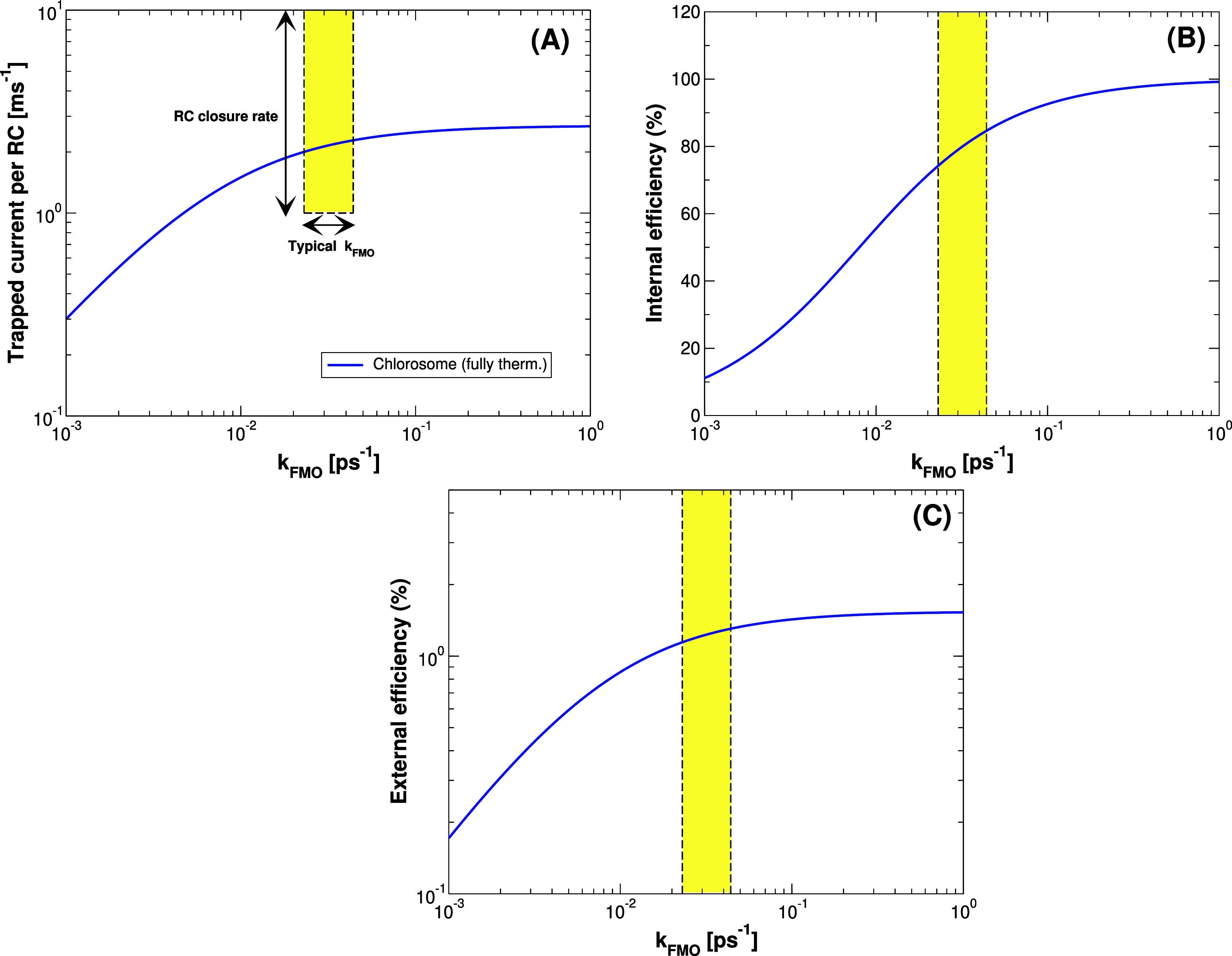
Trapped current, internal and external efficiency vs transfer rate k_FMO_ from baseplate to RCs for the chlorosome coupled to a dimeric baseplate. Trapped current per RC (panel A), internal efficiency (panel B) and external efficiency (panel C) for the entire chlorosome coupled to a dimeric baseplate computed assuming thermalization among all the aggregates. Both the trapped current and the efficiency have been obtained by solving the rate equation system shown in eq 37 at the steady state. The system contains 132840 BChl *c* molecules distributed among three MT adjacent concentric cylinders, each including 44280 BChl molecules, on a baseplate composed of 3350 BChl *a* molecules and with an area of 1145.7 × 3075.3 Å^2^. See Table S3 in Section S4 of the Supporting Information. for more details about the geometry. The yellow box in panels (A–C) between the two vertical dashed lines represents the regime in which FMO complexes typically work (0.023 ps^–1^ < *k_FMO_* < 0.044 ps^–1^). Only for panel A the height of the yellow box represents the range where the RCs closure rate is typically found, which is between 1 and 10 ms^–1^.^23,24^

In Figure 6, the trapped current and the internal efficiency have been computed using the three different approaches described in Section 4 (full rate equations, partially thermalized rate equations, and fully thermalized rate equations) as a function of the *k*_*FMO*_ trapping rate for the single-wall cylindrical models (MT, PD and RD) coupled to a dimeric baseplate. Circular open symbols stand for the full rate equations model (see eqs 26, 27 in Section 4) dashed line for the partially thermalized model (eqs 35 and 36 in Section 4.2) and the continuous line is given by eqs 44 and 45 in Section 4.3, referring to the fully thermalized model.

The first two approaches, full rate equations and partially thermalized rate equations, yield very similar results, see open circles and dashed lines in Figure 6 panels (A–B), providing an accurate description of the excitation energy dynamics. The validity of the partially thermalized rate equations is further supported by the fact that thermalization within these aggregates occurs in few ps, while exciton transfer between aggregates is slower and occurs on a time scale 2 orders of magnitude longer, tens of picoseconds. The fully thermalized model does not provide equivalent results, see the continuous lines in Figure 6. It overestimates the efficiencies and trapped current and it is independent of the specific kind of cylinder considered (MT, PD, RD), see eq 46. However, for the MT model, which exhibits the largest values of trapped current and internal efficiency, the results computed by using this approach are close to the ones obtained by using the full rate equations and the partially thermalized model. Therefore, due to the large size of the biggest aggregate considered here, the chlorosome coupled to a baseplate comprising more than 10^5^ Bchl molecules, only the fully thermalized model has been considered.

Figure 7 shows the trapped current (panel A), the internal efficiency (panel B) and the external efficiency (panel C) as a function of the *k*_*FMO*_ trapping rate for the entire chlorosome obtained by using the fully thermalized model. One of the most interesting results is that the trapped current per RC (see panel A), which is of the order of ∼1 ms^–1^ in the realistic range of values for *k*_*FMO*_, matches the closure rate range of the RCs, see yellow box in Figure 7. This suggests that natural complexes may tend to optimize the number of excitations which arrive on the RC in order to match its operational time. Indeed, a lower number of excitations per second arriving on the RC would leave the RC in the open state for longer times, thus decreasing the rate of charge separation, while a larger number of excitation per second would not be used, since the RC could be in the closed state. In this model the RC closure rate has not been treated dynamically and saturation effects have not been included, since these natural systems operate under low light intensity. In natural biological systems, once charge separation occurs, the RC can no longer undergo excitation, leading to the dissipation of any additional excitation energy. While a full description of the RC dynamics is beyond the scope of this manuscript, it would be certainly interesting in the future to further analyze this issue. Panel B shows the internal efficiency that can reach values between 70 and 85% for the entire chlorosome for 0.023 ps^–1^ < *k*_*FMO*_ < 0.044 ps^–1^, the typical range of FMO (see the yellow window). Note that this value of internal efficiency is in good agreement with refs 16,25,26 On the other hand, the external efficiency (see panel C) has a value close to ∼1% in agreement with our theoretical prediction, see also Section S3 in the Supporting Information.

From previous results shown in Figures 6 and 7, we can understand that accounting for the entire chlorosome is essential to accurately capture the optimal operating conditions of GSB light-harvesting complexes. Indeed, only the chlorosome architecture can process a trapped current per RC that matches the RC closure rate, thereby optimizing the energy transfer process, see panel (A) in Figure 7. Conversely, the single MT cylinder fails to reproduce this optimal condition. Nevertheless, both models, single cylinder and entire chlorosome, yield similar values for the internal efficiency, close to the ones found in literature. A more detailed justification for the necessity of the chlorosome model, along with a systematic comparison to the single cylindrical MT model, is provided in Section S9 of the Supporting Information.

### 5.2 Study of the Relationship between Geometry and Efficient Energy Transfer

In order to explain the better efficiency of natural structures (MT model with a single-wall cylinder and the entire chlorosome) to transfer the excitation to the RCs with respect to the other single-wall cylinder models (RD and PD), we now analyze the dependence of the efficiency on the orientation of the TDMs in the cylinder and the coupling strength between different cylinder models and the baseplate. In Figure 8 (panels (A–B)) the trapped current and the internal efficiency are computed for a single cylindrical aggregate containing 6000 BChl *c* coupled to a dimeric baseplate with 2184 BChl *a* as a function of the TDMs orientation with respect to the cylinder main axis, given by the β angle, see panel D of Figure 8. For β = 0 we have the PD model, for β = 55° we have the MT model^7,36,37,62^ for β = π/2 we have the TD model (tangent dipoles) discussed in ref 36. Panels (A–B) show that among all the possible orientations of the TDMs, for β = 55°, which is the angle found for the MT model (see the red dashed line), the two figures of merit reach the largest values. We also included the RD model (see the gray area between the horizontal black dashed lines), which has large values of both trapped current and internal efficiency, but always smaller than the MT model.

**Figure 8:**
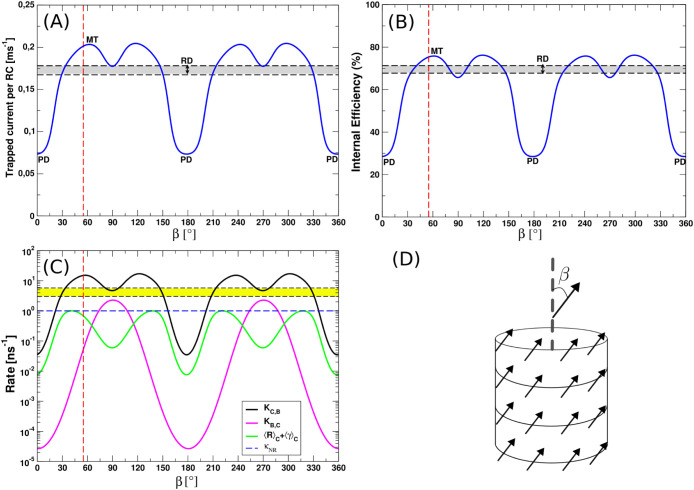
Study of the relationship between trapped current and internal efficiency in single-wall aggregates and the orientation of TDMs. Panels (A–B) show the trapped current and the internal efficiency as a function of the β angle of each TDM and the main axis of the cylindrical aggregate. Each model contains 6000 BChl *c* molecules in the cylinder and 2184 BChl *a* molecules in the baseplate. The results have been obtained by assuming thermalization inside each aggregate, see eqs 35 and 36. The red dashed line represents β = 55°, the typical angle found for the MT model. The gray window between the two black dashed lines represents the interval for the trapped current and efficiency computed for the RD model and averaging over 10 realizations for random dipole orientations. The two black dashed lines in panels (A–B) represent the average value of trapped current and internal efficiency ± one standard deviation. Panel (C): the rates used in eq 30(a–b) have been shown as a function of β angle. The black and magenta curves are the MC-FRET rates from cylinder to baseplate and vice versa, respectively. The green curve is the radiative decay rate from the cylinder, accounting for both fluorescence and stimulated emission due to sunlight. The blue dashed line represents the nonradiative decay rate κ*_NR_*. Finally the yellow window between the black dashed lines represents the average trapping rate from baseplate to RCs κ = 0.138 · *k_FMO_* that ranges from ∼3.17–6 ns^–1^. Panel (D): the orientation of the TDMs in a cylindrical structure is represented. β is the angle between each TDM and the main axis of the cylinder. The positions of the TDM are the same used for the MT model, but we vary continuously the β angle and we keep α, the alternating angle of each dipole with respect to the tangent plane of the cylinder, equal to zero, see ref 36 for more details about the geometry.

A possible explanation for the higher efficiencies of the natural model (MT) can be found analyzing the MC-FRET transfer rates, see Section 4.2: *K*_*C*,*B*_ from cylinder to baseplate and *K*_*B*,*B*_ from baseplate to cylinder. Panel C in Figure 8 shows the MC-FRET rates *K*_*C*,*B*_ and *K*_*B*,*B*_ (black and magenta curves respectively) given by eq 29 and the thermal-averaged emission rate from the cylinder ⟨*R*⟩_*C*_ + ⟨γ⟩_*C*_ (green curve), see eq 33 used in eq 30(a–c) as a function of β angle. From panel C of Figure 8 we can see that the MC-FRET rate from cylinder to baseplate *K*_*C*,*B*_ is faster than the backward rate and the thermal-averaged emission rate, ensuring most of the excitation in the cylinder is funneled to the baseplate. *K*_*C*,*B*_ has the same behavior as the trapped current and the internal efficiency, reaching the largest values for β = 55°, which is about 15 ns^–1^ in agreement with ref 16. Once the excitation reaches the baseplate, it is transferred to the RCs through the FMO complexes by the average FMO trapping rate κ (see the horizontal yellow window), that typically ranges from 3.17 to 6 ns^–1^ in GSB light-harvesting aggregates. Other radiative and nonradiative processes are present in these systems (see the continuous green curve and the blue dashed line respectively), but for the MT model (β = 55°) these quantities are more than 1 order of magnitude less than the MC-FRET rate from cylinder to baseplate.

These findings explain that only specific geometries can exploit an efficient exciton energy transfer under natural sunlight even in the presence of thermal dephasing comparable to room temperature energy and confirm that natural models are the only ones capable to present a geometrical arrangement of TDMs such that both the trapped current and the internal efficiency are maximized with respect to the other mathematical models. Furthermore, the results shown Section S10 in the Supporting Information, where the TDMs are randomly and uniformly distributed in a cone around their original direction, clearly show that the system can maintain a degree of robustness against moderate angular fluctuations, while it is optimized for the β close to 55°.

See also Section S11 in the Supporting Information, where the results for another natural model, the wild type (WT), has been provided and compared to the MT model. The values of the trapped current and internal efficiency found for the WT are close to the ones found for the MT model and higher than all the other mathematical models.

The origin of such a fast MC-FRET rate *K*_*C*,*B*_ in the MT model cylinder is investigated by comparing the spectral properties of the different models (MT, PD and RD) involved in the calculation of *K*_*C*,*B*_. MC-FRET rates describe the incoherent excitation energy transfer from a donor to an acceptor unit. In our case the cylinder plays the role of the donor, while the baseplate is the acceptor unit. As already demonstrated in Section S7 in the Supporting Information, the MC-FRET rate *K*_*C*,*B*_ is strictly related to the Förster rates computed between all the possible pairs of eigenstates of cylinder and baseplate, weighted on the Boltzmann factor for the donor unit (the cylinder). Förster rates given in eq 25 depends on the squared coupling strength between the TDMs associated with the eigenstates of the donor and acceptor units. In Figure 9 the MC-FRET rates *K*_*m*,*BPL*_ = ∑*_n∈BPL_p*_*m*_ *K*_*nm*_ between each eigenstate of the cylinder, indicated by the index *m*, and all the eigenstates of the baseplate is shown for MT, PD and RD models (panels (A–C)) as a function of the dipole strength and the eigenvalues of the cylinder (see eq S64 in Section S7 in the Supporting Information.). The most relevant terms that mainly contribute to the MC-FRET rate *K*_*C*,*B*_ = ∑*_m∈C_K*_*m*,*BPL*_ arise from eigenstates in the energy window between the lowest eigenvalue of the cylinder *E*_1_ and *E*_1_ + *k*_*B*_ *T* (*T* = 300 K), as dictated by the Boltzmann factor shown in eq 29, see the gray window in panels (A-C) between the two dashed lines. Panel A shows that for the MT model the states with the largest MC-FRET rate lie in the lowest part of the spectrum within the gray area. These states dominate the total rate *K*_*C*,*B*_, resulting in an overall fast MC-FRET from the cylinder to the baseplate. Note that not only some superradiant states have a large MC-FRET but also some subradiant states. Indeed for such closeby aggregates it is not the dipole moment of the eigenstates which determined the efficiency of the energy transfer. On the other hand, for the PD model (see panel B) the eigenstates with the highest MC-FRET are far from the gray area. As a consequence, for the PD model MC-FRET is significantly lower than in the MT model. Note that for the PD model the *K*_*m*,*BPL*_ rates are 3 orders of magnitude lower than the ones found for the MT model. Finally for the RD model (panel C) a few eigenstates included in the gray area can still show *K*_*m*,*BPL*_ rate comparable to those ones found for the MT model, however the trapped current and the efficiency are worse (see panels (A–B) in Figure 8).

**Figure 9:**
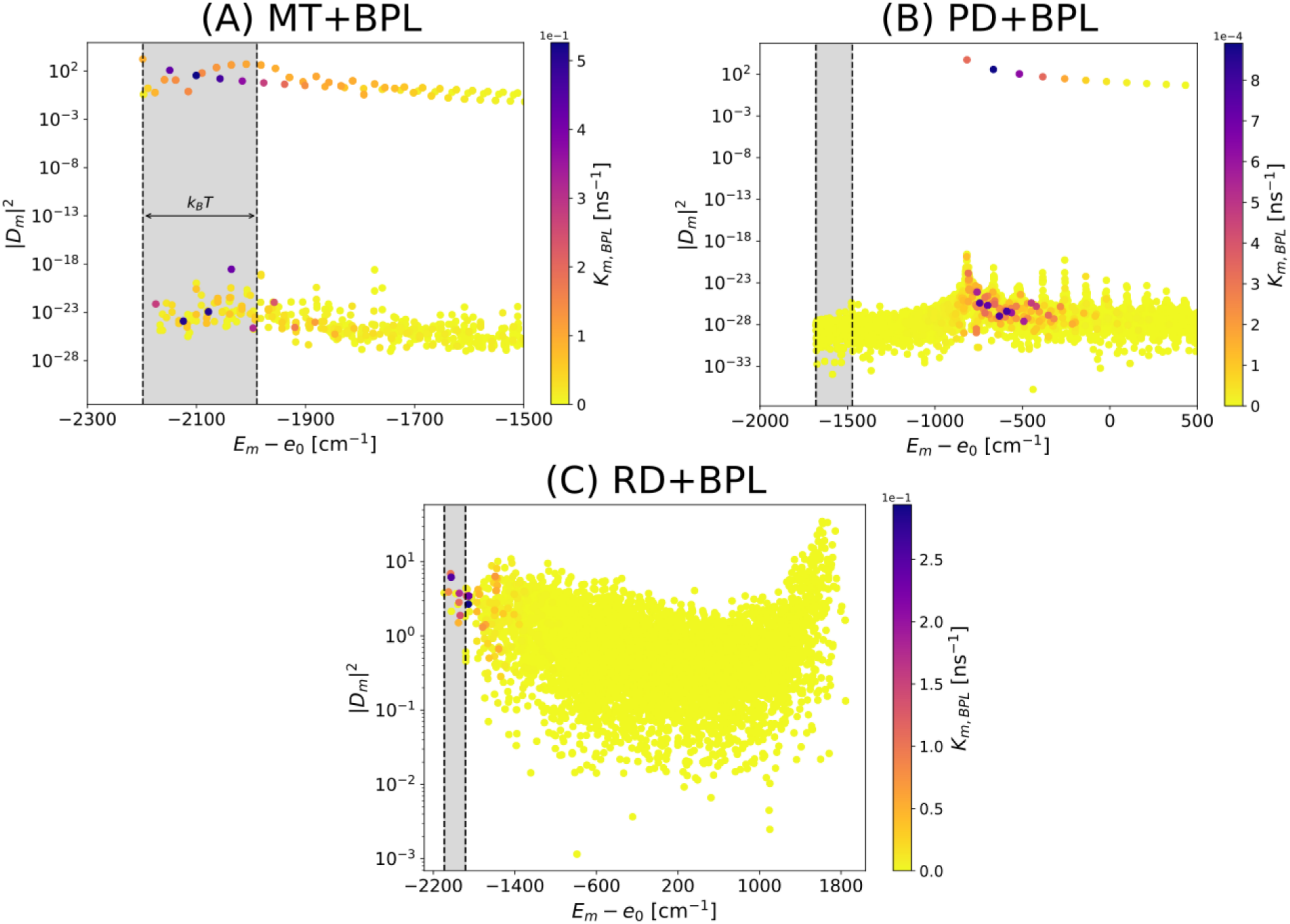
Transfer rates K_m,BPL_ between each eigenstate of the cylindrical models and baseplate. Panels (A–C): transfer rates computed between each single eigenstate of the cylinder and all the eigenstates of the baseplate for MT and PD and RD models coupled to a dimeric baseplate. The rates *K*_*m*,*BPL*_ = ∑*_n∈BPL_p_m_K_nm_* are represented as a function of the dipole strength |*D_m_* |^2^ and the eigenvalues *E_m_*-*e*_0_ of the cylinder (see eq S64 in Section S7 in the Supporting Information). Note that the dipole strength has been represented in logarithmic scale. The system contains 6000 BChl *c* molecules in the cylinder and 2184 BChl *a* molecules in the baseplate. The indices *m* and *n* refers to the eigenstates of the cylinder and baseplate respectively, while *e*_0_ is the excitation energy of BChl *c*. The gray area between the two dashed lines represents the energy region between the lowest eigenvalue *E*_1_ of the cylinder and *E*_1_ + *k_B_T* computed at room temperature (*T* = 300 K). Panels (A–B): *K*_*m*,*BPL*_ rates for MT and PD models, respectively. In these cases only the lowest portion of the spectrum, where the SRSs and the most relevant rates are present, is represented. Here the maximal dipole strength reaches 1760 and 2638 for MT and PD, respectively. Panel (C): *K*_*m*,*BPL*_ rates for a single realization of random dipoles. In this case the whole spectrum has been represented. For RD model the maximal value of the dipole strength is about ∼50.

### 5.3 How Static Disorder Affects Exciton Energy Transfer

Considering the effect of disorder to model the environment is an important issue in light-harvesting complexes. Here we propose a more realistic study of the energy transfer in the single cylindrical aggregates (MT, PD and RD) by adding static disorder. Static disorder is modeled as space-dependent and time-independent fluctuations of the site energies, keeping the couplings between the molecules constant. This approach has been widely used in literature.^16,33,36,37,63,64^ The fluctuations which occur on a time scale much larger than the time scale of the dynamics are usually described as static disorder. Specifically, we consider energy fluctuations that are uniformly distributed around the excitation energy of the molecules *e*_0_, between *e*_0_ – *W*/2 and *e*_0_ + *W*/2, where *W* represents the disorder strength. In this study the trapped current and internal efficiency are computed as a function of static disorder for the three single cylindrical aggregates (MT-PD-RD models) by solving the rate equations given in eq 30 and assuming thermal equilibrium within each aggregate, see Figure 10. Static disorder affects both BChl *a* in the baseplate and BChl *c* in the cylindrical aggregates and the values of current and internal efficiency have been computed averaging over 100 realizations of static disorder for MT and PD models and 10 × 10 realizations of disorder and random orientations of TDMs for the RD model, respectively. Figure 10 shows the trapped current (panel A) and internal efficiency (panel B) as a function of the disorder strength for the single cylinder models (MT, PD and RD) comprising 6000 BChl *c* coupled to a dimeric baseplate with 2184 BChl *a*. The main results found in Figure 10 demonstrate that all the models (MT, PD and RD) are robust to static disorder, showing constant values of both trapped current and internal efficiency up to a disorder strength *W* close to 10^3^ cm^–1^. For larger values of *W* both figures of merit in MT and RD models start to drop, while for the PD model they incre­[ase, reaching a common value for all the models. The yellow window between the two black dashed lines represents the realistic values of static disorder found in the literature^16,26,32,65,66^ for GSB light-harvesting complexes (1014 cm^–1^ < *W* < 1368 cm^–1^).

**Figure 10:**
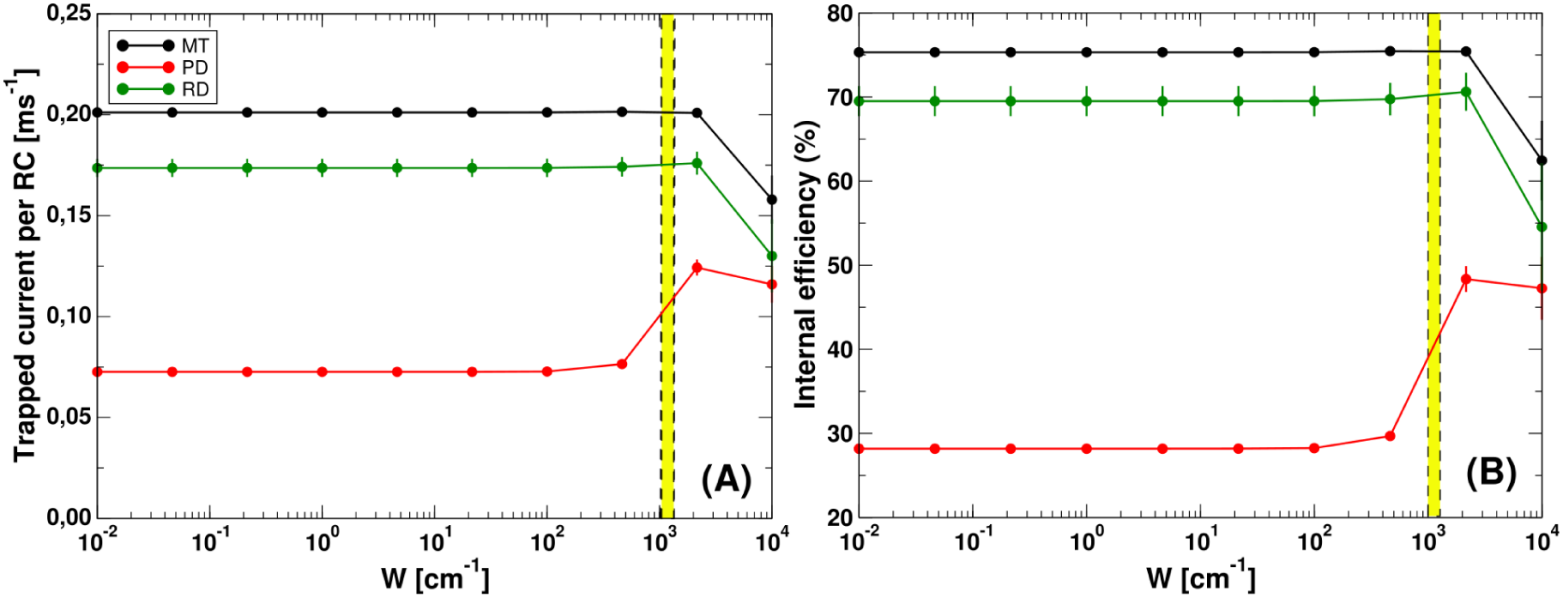
Static disorder in single cylindrical complexes coupled to a dimeric baseplate: trapped current and internal efficiency. Panels (A–B): trapped current per RC and internal efficiency as a function of static disorder strength *W* for all the single cylinder models MT, PD and RD comprising 6000 BChl *c* coupled to a dimeric baseplate with 2184 BChl *a*. In all panels all the simulations have been run by assuming thermalization inside each aggregate. Equations 35 and 36 in the main text have been used to compute respectively the trapped current per RC and the internal efficiency. Numerical results have been obtained by averaging over 100 realizations for MT and PD models, while for the RD model 10 × 10 realizations have been done by changing both the randomness and the disorder strength. The yellow window between the two dashed vertical lines represent the typical disorder strength found in literature for GSB light-harvesting systems (1014 cm^–1^ < *W* < 1368 cm^–1^).^16,26,32,65,66^

## 6 Conclusions

In this manuscript we have studied the excitation energy transfer process in the entire light-harvesting apparatus of GSB, comprising more that 100000 BChl molecules distributed in the chlorosome and in the baseplate, from solar light absorption to the trapping of excitation in the RCs. Sunlight is modeled through its blackbody spectrum, taking into account the Earth–Sun distance. Also the coupling to room temperature thermal bath in the presence of static disorder has been taken into account. We have developed three rate equations approaches in order to describe the process of the energy transfer: full rate equations model, with the highest computational costs, partially thermalized and fully thermalized models. The partially thermalized model requires less numerical efforts and it gives results in agreement with the full rate equation approach. All the approaches used to model excitation energy transfer (full rate equations, partially thermalized and fully thermalized rate equations) rely on the secular approximation and the assumption that the effects of multiple environments, the electromagnetic field and the phonon bath, can be treated independently in the Lindblad master equation. The problem of the interplay of multiple environments is very delicate, ref 67: both the assumption that the environments can be treated independently and the secular approximation, might not be justified in the presence of resonances overlapping. Indeed, the secular Redfield is valid if the energy level spacing is considerably larger than the Redfield rate. If this condition is not obeyed, one can use the full Redfield equation without the secular approximation for the nearly degenerate states, see ref 68. Ideally, one could avoid secular approximation in the subspace of the closely spaced states and apply it outside the subspace. In perspective it would be very useful to analyze further how the interplay of different environments affects energy transfer in antenna complexes.

The chlorosome has been modeled as three adjacent concentric cylindrical aggregates coupled to a dimeric baseplate. The trapped current per RC and the internal efficiency have been chosen as the two main figures of merit and we have demonstrated that for the chlorosome coupled to the dimeric baseplate the trapped current matches the RC closure rate, while the internal efficiency is about ∼80%. In order to investigate the high efficiency of natural systems, we have considered smaller systems composed of a single wall cylindrical aggregate coupled to a baseplate: the MT model, used to build up the entire chlorosome, and other mathematical models, obtained by changing the β angle of each TDM with respect to the main axis of the cylinder. In particular we focused our study on two mathematical models: the RD model, where all the TDMs have random orientation in the space, and the PD model, where all the TDMs are parallel to the cylinder axis (β = 0°). The main foundings presented in this paper confirm that natural models show the largest values of trapped current and internal efficiency and their behavior is strictly related to their geometry. In fact, natural models (single cylinder MT model and the entire chlorosome coupled to the dimeric baseplate) are characterized by the typical orientation of their TDMs with respect to the cylinder axis (β = 55°), that ensures a fast MC-FRET from the cylinder to the baseplate, faster than all the other radiative and nonradiative processes.

The emergence of fast energy transfer only in the natural models has been investigated by studying the MC-FRET from each single cylinder eigenstate to the baseplate *K*_*m*,*BPL*_, where *m* stands for an eigenstate of the cylinder. The main results show that only the MT model can support rapid MC-FRET rates within the *k*_*B*_ *T* energy region at room temperature. These fast transfer rates correspond to both superradiant states of the cylinder and subradiant ones, revealing that for such closely spaced aggregates, energy transfer efficiency is not determined by the net dipole moment of the eigenstates alone. To determine the general conditions which allow efficient energy transfer in natural models remains a pivotal question in quantum biology and represents a compelling direction for future research.

Our results demonstrate the non trivial interplay of geometry and functionality in realistic light-harvesting systems, showing that the specific symmetry present in natural complexes is optimal for energy transfer. It should be noted, however, that natural architectures are subjected to complex environmental and conformational constraints. While we have addressed random dipoles and site energy disorder here, demonstrating the robustness of the natural model to the presence of static disorder comparable to room temperature energy and identifying a tolerance range for dipole orientations, exploring correlated positional and orientational disorder remains an interesting direction for future perspective. Proving the sensibility of energy transfer on the specific dipole disposition and orientation, our analysis will inspire the design of artificial light-harvesting systems.

## Supplementary Material



## Notes

Solar irradiance; optical properties of BChl *a* and *c;* external efficiency of the GSB light-harvesting complex; table of the models; regime of validity of the Hamiltonian models; general derivation of Lindblad Master Equations for independent phononic baths; multichromophoric transfer rates MC-FRET; parameters of the model; size-dependent energy transfer efficiency and trapped current in GSB light-harvesting architectures; analysis of TDMs distribution in a cone; comparison between MT and WT models (PDF)
