# Supplementary material for: Relation between Structure and Functionality in Photosynthetic Antenna Complex of Green Sulfur Bacteria: Efficiency under Natural Sunlight Pumping
Source: J Phys Chem B. 2026 May 15;130(21):5260–77. doi: 10.1021/acs.jpcb.6c00303 (PMC13224166; doi:10.1021/acs.jpcb.6c00303)
Supplement: Supplementary file 1 [file jp6c00303_si_001.pdf]

Supporting Information: Relation between Structure and Functionality in Photosynthetic Antenna Complex of Green Sulfur Bacteria: Efficiency under Natural Sunlight Pumping

Alessia Valzelli,[*,†,‡,¶] Francesco Mattiotti,[§] Jianshu Cao,[‖] and Giuseppe Luca Celardo[‡,¶,⊥]

†*Dip. di Ingegneria dell'Informazione, Univ. degli Studi di Firenze, 50139 Firenze, Italy*

‡*Dip. di Fisica e Astronomia, Univ. degli Studi di Firenze e CSDC, 50019 Sesto Fiorentino, Italy*

¶*Istituto Nazionale di Fisica Nucleare, Sezione di Firenze, 50019 Sesto Fiorentino, Italy*

§*Theoretische Physik, Universität des Saarlandes, D-66123 Saarbrücken, Germany*

‖*Department of Chemistry, Massachusetts Institute of Technology, 77 Massachusetts Avenue, Cambridge, Massachusetts 02139, USA*

⊥*European Laboratory for Non-Linear Spectroscopy (LENS), Univ. degli Studi di Firenze, 50019 Sesto Fiorentino, Italy*

E-mail: alessia.valzelli@unifi.it

S1 Solar irradiance

In this section a study of the solar irradiance has been given by using different approaches. The solar spectrum measured on the Earth is analyzed and compared with the Planck's law for a black body at a finite temperature $T_S = 5800$ K, typical of sunlight. In reality, the sunlight radiation is not exactly described by black-body radiation, as the photon experiences multiple scatterings upon arriving on the earth. Yet, the measured spectrum of sunlight is sufficiently broad compared with the absorption spectrum of light-harvesting systems (see the absorption spectra of BChl molecules in Sec. S2.2), such that the predictions of our model calculation are not sensitive to the precise functional form of the sunlight spectrum.[S1]

From the black body theory, sunlight radiation is modeled as a photon bath at a given temperature T_S. Due to the fact that photons are boson particles, they can be studied with the Bose-Einstein statistics, that describes how a collection of non-interacting identical particles may occupy a set of available discrete energy levels at thermodynamic equilibrium. Here this approach has been used and the occupation number of photons at a given frequency ω and temperature has been determined. In this case the Bose-Einstein distribution $n_S(\omega)$ reads:

$$n_S(\omega) = \frac{1}{e^{\hbar\omega/(k_B T_S)} - 1}. \tag{S1}$$

Fig. S1 shows the comparison between experimental and theoretical models for solar irradiance as a function of the wavelength. The function here represented by the black line is the well known Planck's law at finite temperature T_S multiplied by a coefficient Ω_S, see Eq. (S2), that accounts for the angle under which the Sun can be seen from Earth. The Planck's law for a black body at a temperature T_S at a given wavelength λ reads:

$$g(\lambda, \Omega_S) = \Omega_S \frac{2hc^2}{\lambda^5} \frac{1}{\exp\left(\frac{hc}{k_B T_S \lambda}\right) - 1}, \tag{S2}$$

where h is the Planck's constant, c the speed of light, k_B the Boltzman's constant and Ω_S

the solid angle under which the Sun is seen from the Earth.

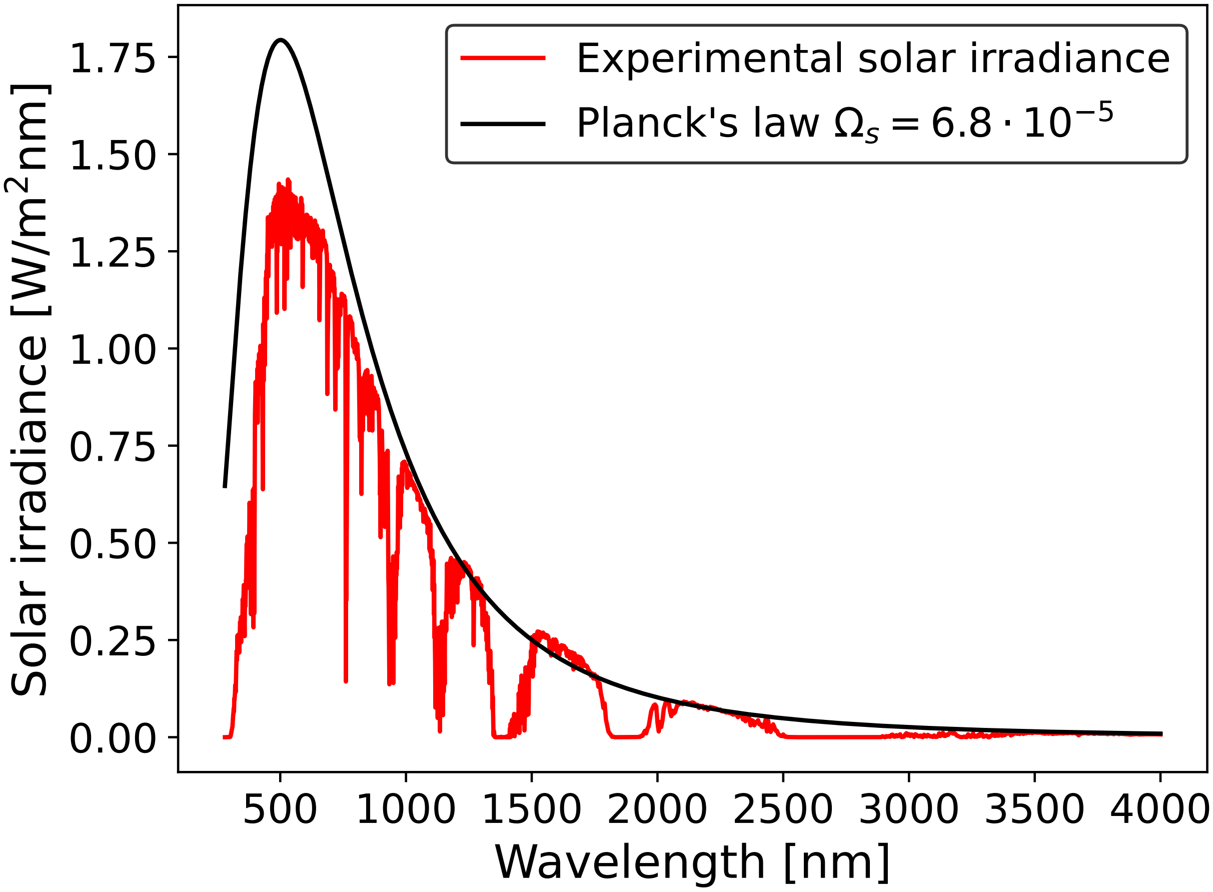

Figure S1: *Solar irradiance: comparison between experimental data measured on the Earth surface and black body theory.* Comparison between an experimental solar spectrum (red continuous line) and the Planck's law $g(\lambda, \Omega_S)$ (black continuous line, see Eq. (S2)) for a black body at the Sun temperature $T_S = 5800$ K multiplied by $\Omega_S = 6.8 \cdot 10^{-5}$, the solid angle under which Sun is seen from the Earth.

In Ref. S2 the concentration of the solar radiation on the Earth has already been computed. Viewed from the Earth, the Sun has an angular diameter of $\alpha_S = 0° \, 32'$, corresponding to a solid angle Ω_S given by

$$\Omega_S = 2\pi \int_0^{\alpha_S/2} \sin(\theta) \, \mathrm{d}\theta = 6.8 \cdot 10^{-5} \ . \tag{S3}$$

Because of the small value of the solid angle Ω_S, the sunlight energy current density measured on the Earth is reduced with respect to the total energy flux emitted by the Sun.

Integrating the experimental solar irradiance $\Phi(\lambda)$ (red curve in Fig. (S1)), we obtain $P_{Sun} = \int_{\lambda_i}^{\lambda_f} \Phi(\lambda) d\lambda = 901.24$ W/m^2 (with cut-offs $\lambda_i = 280$ nm and $\lambda_f = 4000$ nm), which is close to 1353 W/m^2 found in Ref. S2 referring to the solar irradiance measured outside the atmosphere. The small discrepancy between the two data is due to the Earth atmosphere and it strongly depends on the latitude, the weather conditions and the incident angle of sunlight with respect to the normal to the Earth surface.

Our findings show that modeling sunlight through the black body radiation is a good approximation for sunlight. The spectrum computed using the black body theory can be compared to the experimental spectrum, minus a factor Ω_S that takes the Sun-Earth distance into account. This approach has already been done in literature. In Ref. S3 the Sun is seen as a black body at finite temperature T_S. The authors used the parameter f_S, which represents the fraction of solid angle under which Sun is seen from Earth, to compute the occupation number of photons at a given wavelength and temperature. f_S found in Ref. S3 is related to the solid angle Ω_S by the following relationship:

$$f_S = \Omega_S / 4\pi = \frac{\pi r_s^2}{4\pi R_{ES}^2} = 5.4 \cdot 10^{-6}, \tag{S4}$$

depending on the radius of the Sun r_s and the Sun-Earth distance R_{ES}.

In reality, the sunlight radiation is not exactly described by black-body radiation, as the photon experiences multiple scatterings upon arriving on the earth. Yet, the measured spectrum of sunlight is sufficiently broad compared with the absorption spectrum of light-harvesting systems (see the absorption spectra of BChl molecules in Sec. S2.2), such that the predictions of our model calculation are not sensitive to the precise functional form of sunlight spectrum.[S1]

S2 Optical properties of BChl *a* and *c*

In this section the optical properties (emission and absorption) for BChl *a* and *c* molecules typical of GSB and PB light-harvesting complexes are computed by using different methods.

First a subsection for the emission rate is given. Then the absorption rate is computed through the black body approach, where each BChl molecule is seen as a TLS and only the main absorption transition frequency and its transition dipole moment are taken into account. Then a more accurate estimation of the absorption is given by integration of the overlap between the absorption cross section and the solar irradiance for all the transition frequencies.

S2.1 Emission

The radiative decay rate of a molecule, modeled as a TLS, can be estimated as the inverse of the fluorescence lifetime τ_{fl} and reads:

$$\frac{\gamma}{\hbar} = \frac{1}{\tau_{fl}} = \frac{4\mu_0^2\omega_0^3}{3\hbar c^3}, \tag{S5}$$

where $\omega_0 = \frac{2\pi c}{\lambda_0}$ is the transition frequency, while μ_0 is its corresponding transition dipole moment. Tab. S1 shows all the parameters for BChl *a* and *c*.

Table S1: *Emission in BChl molecules.* **The table shows the emission parameters for BChl *a* and *c* molecules found in literature.[S5–S8]**

	BChl *a*	BChl *c*
TDM μ_0 [Debye][S4]	10	5.6
Trans. wavelength λ_0 [nm]	780	670
$\gamma/\hbar$ [s^{-1}]	$7 \cdot 10^7$	$3.2 \cdot 10^7$
τ_{fl} [ns]	15	30

S2.2 Absorption

Black body theory Here we compute the absorption rate for BChl a and c through the black body theory and assuming that each BChl molecule can be treated as a TLS with excitation energy e_0 and TDM $\vec{\mu_0}$, given in Tab. S1. We can express the absorption rate as follows,

$$P_{abs} = \frac{\gamma}{\hbar} n_S(\omega) f_S e_0 \ , \tag{S6}$$

where $e_0 = hc/\lambda_0$ is the excitation energy of single BChl molecules (see Tab. S1 for the values of λ_0 for BChl a and c), $n_S(\omega)$ represents the occupancy of photons at the Sun temperature $T_S = 5800$ K for a given frequency ω (see Eq. (S1)), while f_S is the fraction of solid angle from which sunlight is seen from the Earth.

Using the parameters shown in Tab. S1, the absorption rate for BChl a and c is computed, showing good agreement with the data found in literature:[S8]

- BChl a: $P_{abs} = 4 \cdot 10^{-18}$ W, corresponding to $N_{abs} = 15.8$ s^{-1} photons per second;
- BChl c: $P_{abs} = 1.3 \cdot 10^{-18}$ W, corresponding to $N_{abs} = 4.6$ s^{-1} photons per second.

Absorption cross section Refs. S7,S9–S12 report the extinction coefficient ϵ for BChl molecules in units of M^{-1}cm^{-1}. Through an easy calculation already found in Ref. S8, the absorption cross section in cm^2 can be obtained as

$$\sigma = \frac{\ln(10)\epsilon c_m}{n} = \frac{\ln(10)10^3\epsilon}{N_{av}}, \tag{S7}$$

where c_m and n are respectively the concentration of the solution in molarity [M] and in [cm^{-3}], N_{av} is the Avogadro's number and $c_m = n\frac{10^3}{N_{av}}$ is the relationship between c_m and n.

Fig. S2 shows the solar irradiance $\Phi(\lambda)$ (black line) and the absorption cross section for both BChl a (σ_a in red line) and c (σ_c in blue line).

Finally the number of photons absorbed by each BChl has been computed by computing

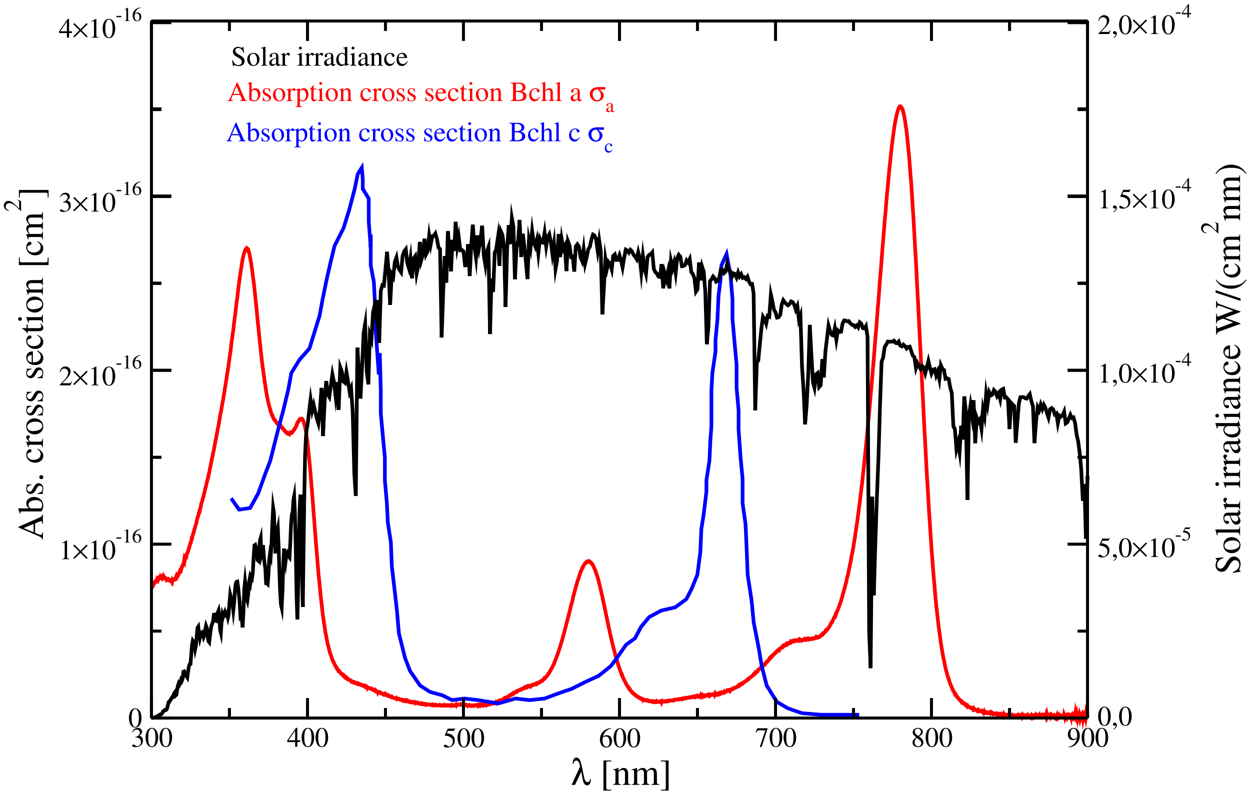

Figure S2: *Solar irradiance and absorption cross section for BChl a and c.* Solar irradiance (black line) and absorption cross section for BChl *a* (red line) and *c* (blue line) are represented as a function of the transition wavelength. The absorption cross section has been computed by Eq. (S7) from experimental data of the extinction coefficient found in literature.[S7,S9–S12]

numerically the following integral:

$$N_{abs} = \int_{\lambda_i}^{\lambda_f} d\lambda\, \Phi(\lambda)\, \sigma_{a,c}(\lambda)\, \frac{\lambda}{hc}\ . \tag{S8}$$

The number of photons absorbed by a BChl is given by

- $N_{abs} = 12.5\ \mathrm{s}^{-1}$ for BChl a
- $N_{abs} = 8.6\ \mathrm{s}^{-1}$ for BChl c

in good agreement with the previous calculations obtained by using the black body approach and the experimental data reported in literature.[S8]

S3 External efficiency of the GSB light-harvesting complex

Here we compute the external efficiency for the entire light-harvesting complex of GSB, comprising the chlorosome and the baseplate. Chlorosomes contain mainly BChl c, while the baseplate is formed by BChl a molecules. We assume that the photons flux on Earth from sunlight is

$$I_{Sun} = \int_{\lambda_i}^{\lambda_f} d\lambda\, \Phi(\lambda)\, \frac{\lambda}{hc} = 1.75 \cdot 10^{21}\ \mathrm{s}^{-1}\mathrm{m}^{-2}\ , \tag{S9}$$

obtained by integration of the solar irradiance given in Fig. S2 from $\lambda_i = 280$ nm to $\lambda_f = 4000$ nm and in agreement with the results provided in Ref. S8. Using the results found in the previous sections, the total absorption rate in the GSB light-harvesting system is computed. The total absorption rate in the chlorosome and in the baseplate is given respectively by:

- $N_{chl} = 132840$ BChl c and $N^c_{abs} = 132840 \cdot 8.6 = 1184299\ \mathrm{s}^{-1}$,
- $N_{BPL} = 3350$ BChl a and $N^a_{abs} = 3350 \cdot 12.5 = 41875\ \mathrm{s}^{-1}$,

where 3350 and 132840 are the total number of BChl molecules in the baseplate and in the chlorosome respectively. If we assume the baseplate area $A_{BPL} = 3.5 \cdot 10^{-14}$ m^2, the absorption rate per unit area in the GSB reads:

$$I_{abs} = \frac{N^c_{abs} + N^a_{abs}}{A_{BPL}} = 3.4 \cdot 10^{19} \text{ s}^{-1}\text{m}^{-2}. \tag{S10}$$

Finally the absorption efficiency for the GSB can be estimated as:

$$\eta_{abs} = \frac{I_{abs}}{I_{Sun}} = 1.9\%. \tag{S11}$$

For light-harvesting complexes three main quantities have been determined: the internal efficiency $\eta_{int} = \frac{I_{RCs}}{I_{abs}}$, the external efficiency $\eta_{ext} = \frac{I_{RCs}}{I_{Sun}}$ and the absorption efficiency η_{abs} defined above in Eq. (S11). In our theoretical model we have found values of the internal efficiency between $70\% < \eta_{int} < 85\%$ for the chlorosome, close to the values found in literature.[S13] Assuming the absorption efficiency is about $\sim 1.9\%$ for the chlorosome, the external efficiency has been determined by

$$\eta_{ext} = \eta_{abs} \times \eta_{int} \tag{S12}$$

and we found values between $1.3 - 1.6\%$.

These calculations represent an approximate estimate of the external efficiency in the GSB antenna complexes in good agreement with the numerical results found by using the rate equations approach. Fig. S3 shows the external efficiency obtained by using Eq. (25) in the main text as a function of the k_{FMO} trapping rate for the chlorosome (see the blue continuous line). The results show that the theoretical prediction given above is in agreement with numerical simulations, considering realistic values for the k_{FMO} trapping rate represented in the yellow window in Fig. S3. Fig. S3 also shows the external efficiency computed by numerical simulations for all the three single cylinder models (MT, PD and RD) coupled to

the baseplate. Here systems formed by a single cylindrical aggregate with 6000 BChl c and a length of 821.7 Å and a dimeric baseplate with 2184 BChl a and an area of 2739.1×550.8 Å^2 have been considered. For single cylinder aggregates lower values of the external efficiency have been found. Also these numerical results can be compared to the theoretical prediction presented in Tab. S2, where a calculation of the external efficiency in the systems formed by a single cylindrical aggregate has been done following the same approach used for the chlorosome.

The total absorption rate in the cylinders and in the baseplate are given respectively by:

- $N^c_{abs} = 51600$ s^{-1},
- $N^a_{abs} = 27300$ s^{-1}.

The absorption rate per unit area in all the models (MT, PD and RD) is given by Eq. (S10) and reads $I_{abs} = 5.26 \cdot 10^{13}$ s^{-1}m^{-2}, while the absorption efficiency is around $\eta_{abs} \approx 0.3\%$ for MT, PD and RD models. The following table shows the external efficiency calculated for the systems comprising a single cylinder and a baseplate.

Table S2: Internal and external efficiencies computed for the single cylindrical models (MT, PD and RD) with 6000 BChl c and a length of 821.7 Å placed above a dimeric baseplate with with 2184 BChl a and an area of 2739.1×550.8 Å^2. The internal efficiency has been taken from Fig. 6 in the main text assuming 0.023 ps^{-1} $< k_{FMO} < 0.044$ ps^{-1}, while the external efficiency has been computed following Eq. (S12).

Model	Int. Efficiency	Ext. Efficiency
MT + BPL	70% – 80%	0.21% – 0.24%
PD + BPL	25% – 30%	0.08% – 0.09%
RD + BPL	65% – 73%	0.2%

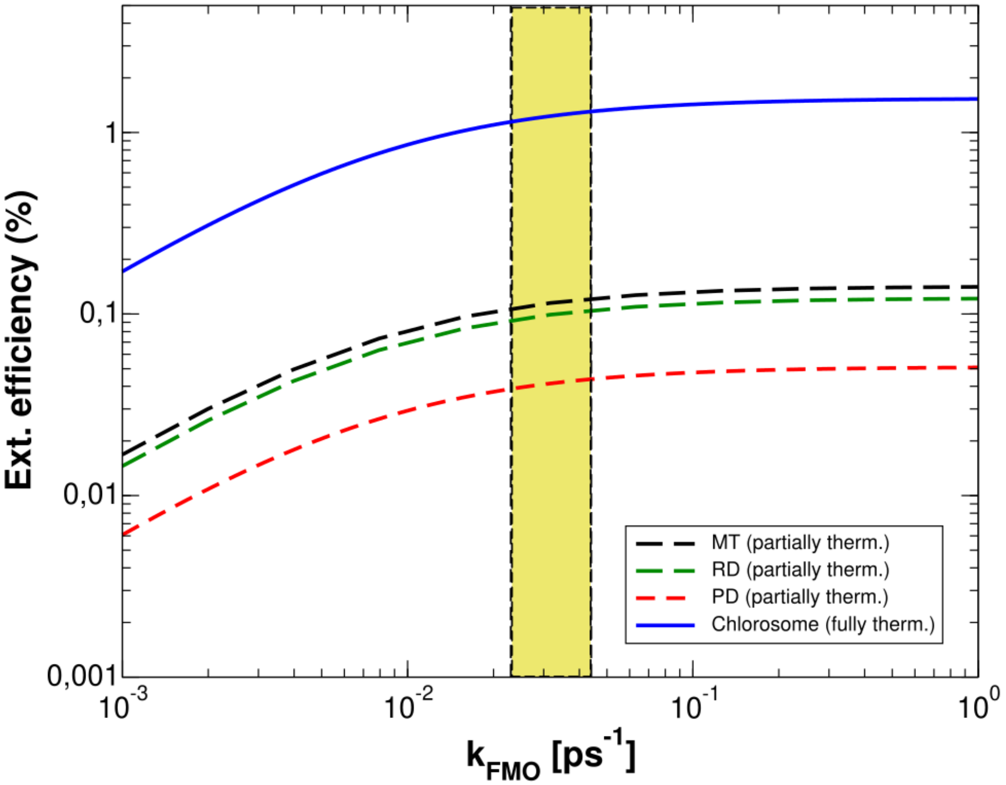

Figure S3: *External efficiency in the chlorosome and in the single cylinder models coupled to a dimeric baseplate.* The figure shows the external efficiency as a function of the k_{FMO} trapping rate for different systems: the chlorosome and single cylinder models (MT-PD-RD) coupled to a baseplate. The results for the entire chlorosome (blue continuous line) have been obtained by assuming thermalization among all the aggregates, while for the single cylinders model the partially thermalized rate equations approach has been used (see the black, red and green dashed lines). For the RD model an average over 10 realizations of random TDMs orientations has been computed. The yellow window represents the typical range for k_{FMO} trapping rate.

S4 Table of the models

Here a table comprising all the models taken into account in this manuscript is given. Tab. S3 contains the total number of molecules and the dimensions for both cylindrical aggregates and baseplate.

Table S3: *Cylinder and dimeric baseplate parameters.* The table shows the sizes and the total number of BChl molecules in each aggregate (single cylinder models (MT, PD, RD and WT) and the entire chlorosome coupled to a baseplate). N_{TOT} is the total number of molecules in the cylinder or baseplate. L_{cyl} refers to the cylinders length, while L_z and L_x are respectively the length and the width of the dimeric baseplate. N_z and N_x represent the number of dimers of the baseplate along the *x* and *z* directions. For the baseplate the total number of BChl molecules is given by $N_{TOT} = 2 \times N_x \times N_z$. Ref. S14 has been taken into account to determine the relative dimensions between cylindrical aggregates and the dimeric baseplate.

MODEL	CYLINDER PARAMETERS	BASEPLATE PARAMETERS
MT/PD/RD + BPL	N_{TOT}=6000<br>L_{cyl}=821.7 Å	N_{TOT}=2184<br>N_Z=91 L_Z=2739.1 Å<br>N_X=12 L_X=550.8 Å
WT + BPL	N_{TOT}=6000<br>L_{cyl}=618,75 Å	N_{TOT}=1608<br>N_Z=67 L_Z= 2016.7 Å<br>N_X=12 L_X= 550.8 Å
CHLOROSOME + BPL	N_{TOT}=132840<br>L_{cyl}=1485.7 Å	N_{TOT}=3350<br>N_Z=67 L_Z=3075.3 Å<br>N_X=25 L_X=1147.5 Å

S5 Regime of validity of the Hamiltonian models

In this section a deeper study of the three Hamiltonian models used in the main text has been addressed (see discussion in Sec. 3 in the main text). In particular here we have studied the regime of validity of three Hamiltonian models (DH, HH and NHH) and we have compared our results of the dipole strength and radiative decay width obtained diagonalizing the three Hamiltonian models with the expectation value of the non-Hermitian part of the full Hamiltonian (NHH), according to the perturbation theory.

As already described in the main text, the perturbed Hamiltonian is given by the NHH model and it reads:

$$\hat{H}_{NHH} = \sum_{i=1}^{N} e_0 |i\rangle\langle i| + \sum_{i\neq j} \Delta_{ij} |i\rangle\langle j| - \frac{\mathrm{i}}{2} \sum_{i,j=1}^{N} Q_{ij} |i\rangle\langle j| \,, \tag{S13}$$

where $e_0 = \hbar\omega_0$ is the excitation energy of the single emitter, while Δ_{ij} and Q_{ij} are the out-of diagonal terms with $Q_{ij} \ll \Delta_{ij}$.

The diagonal part of Δ_{ij} and Q_{ij} are given by:

$$\Delta_{jj} = 0 \,, \qquad Q_{jj} = \frac{4}{3}\mu^2 k_0^3 = \gamma \,, \tag{S14}$$

where γ is the radiative decay width associated to each emitter.

The non-Hermitian Hamiltonian gives complex eigenvalues $\varepsilon_n = \mathrm{E}_n - \mathrm{i}\frac{\Gamma_n}{2}$ where $\Gamma_n/\hbar$ is the radiative decay rate of the nth eigenstate in s^{-1}.

In our systems the out-of diagonal term Q_{ij} can be treated as a perturbation, since $Q_{ij} \ll \Delta_{ij}$. Let us define the unperturbed Hamiltonian, which coincides with the Hermitian part of the NHH shown in Eq. (S13) and reads:

$$\hat{H}_{HH} = \sum_{i=1}^{N} e_0 |i\rangle\langle i| + \sum_{i\neq j} \Delta_{ij} |i\rangle\langle j|, \tag{S15}$$

whose unperturbed eigenvalues and eigenstates are E_n^0 and $|E_n^0\rangle$, respectively. [1]

According to the non-degenerate perturbation theory, the first-order energy shifts [2], which are the expectation values of the perturbation Hamiltonian Q_{ij} while the system is in the unperturbed eigenstate $|E_n^0\rangle$, have been computed and compared to the radiative decay widths obtained by diagonalizing the full non-Hermitian Hamiltonian.

Fig. S4 shows the maximal dipole strength obtained diagonalizing the DH (black circles) and HH (red squares) models and the maximal decay width (blue stars) obtained with the NHH model as a function of the length of the MT cylinder rescaled over λ_0 (see Tab. S1 for the value of λ_0 for BChl *c*). Finally the first-order energy shifts $\langle E_n^0|Q|E_n^0\rangle/\gamma$ (magenta diamonds) computed with the perturbation theory are compared with the previous results. In all models we added a source of static disorder ($W = 1$ cm^{-1}, a single realization), such that degeneracies have been broken while superradiance is preserved. The geometrical model that we consider is the single-wall MT cylinder with 6 TDMs on each ring with a length L that goes from 74.7 to 8291.7 Å.

In Fig. S4 the maximal dipole strength computed with the DH and HH models increases linearly with the length of the system (see black circles and red squares in Fig. S4), while the radiative decay width (see blue stars) tends to saturate. Finally a comparison with the perturbation theory is provided. Since the imaginary part Q_{ij} is a small perturbation of the non-Hermitian Hamiltonian, the first-order energy shifts have been computed as the expectation value of Q_{ij} with the unperturbed eigenstates $|E_n^0\rangle$ of the Hermitian Hamiltonian given by Eq. (S15). The results show that the maximal value of $\langle E_n^0|Q|E_n^0\rangle/\gamma$ (see magenta diamonds in Fig. S4) is in agreement with all the other three methods (DH, HH, NHH) when $L < \lambda_0$. When the system size increases the dipole strength description is no longer

[1] Note that only in this section we introduce this notation to indicate eigenvalues and eigenstates of the unperturbed Hamiltonian (HH), while in the main text we use E_n and $|E_n\rangle$. The reason of this choice is to distinguish clearly between the eigenstates and eigenvalues of the perturbed and unperturbed Hamiltonians.

[2] Note that here we use the non-degenerate perturbation theory to compute the energy-shift. This approach is valid only when the eigenstates of the unperturbed system are non-degenerate. In this case the unperturbed system shows degeneracies that have been broken by adding static disorder $W = 1$ cm^{-1}. Such value of static disorder does not affect the spectrum and does not destroy superradiance, but it is enough to remove the degeneracy.

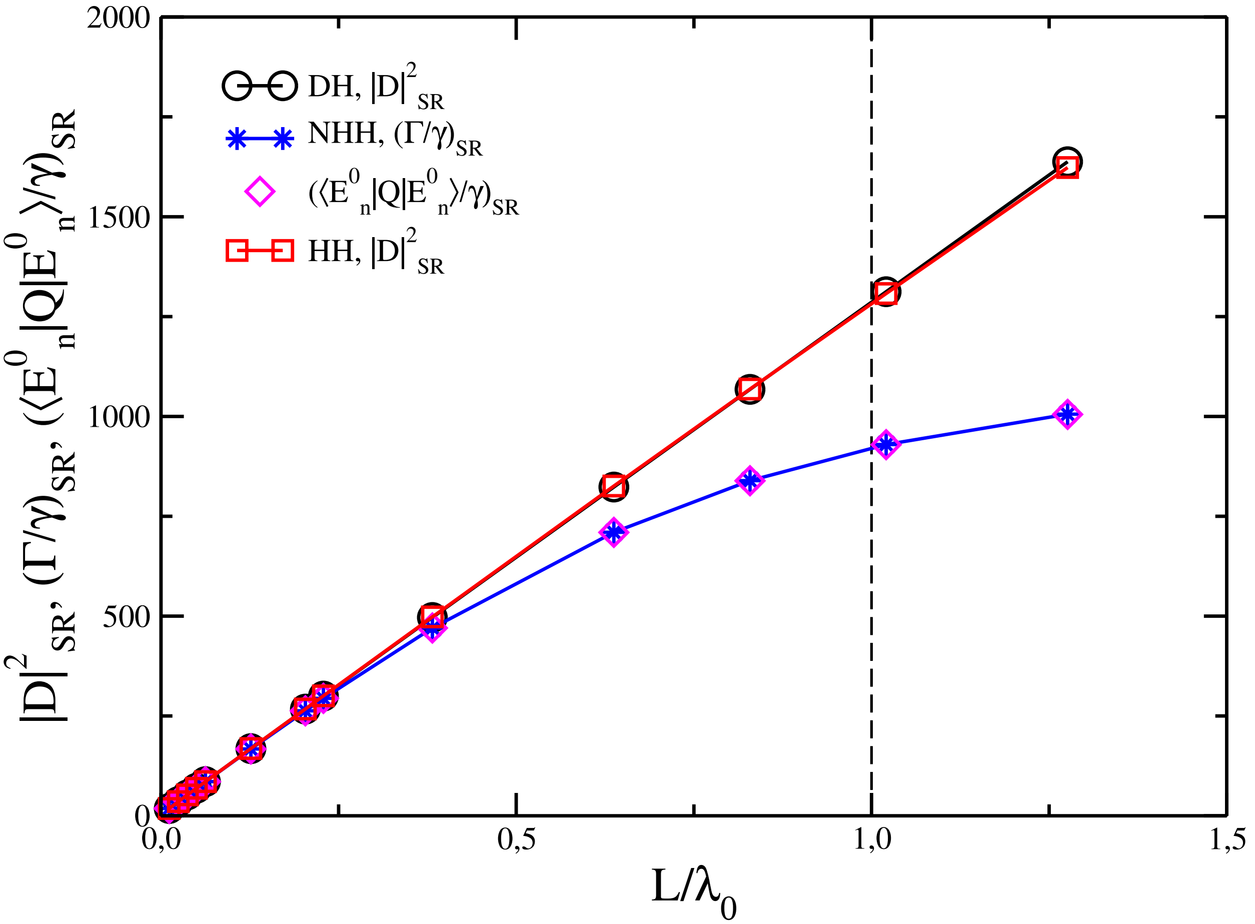

Figure S4: *Regime of validity of the three hamiltonian models and comparison with the perturbation theory approach in a single wall MT cylinder.* Maximal dipole strength obtained by diagonalizing the DH (black cirlces) and HH (red squares) models and the maximal decay width (blue stars) obtained with the NHH model as a function of the length of the MT cylinder rescaled over λ_0. The first-order energy shifts $\langle E_n^0|Q|E_n^0\rangle/\gamma$ (magenta diamonds) computed with the perturbation theory is shown. The geometrical model considered is the single wall MT cylinder with 6 TDMs on each ring with a length $74.7 \leq L \leq 8291.7$ Å. The total number of TDMs goes from 60 to 60000 for the longest cylinder that we consider here. The vertical dashed line ($L = \lambda_0$) stands for a cylinder length comparable to the excitation wavelength of the single emitter. In all models a source of static disorder ($W = 1\text{cm}^{-1}$, a single realization) has been added in order to break the degeneracies in the unperturbed system, without changing the spectral properties of the system and the superradiance response.

valid, while the perturbation approach is still in agreement with the NHH model, showing a tendency of the superradiant decay width to saturate with the length of the cylinder.

From our analysis, we can conclude that all the models (dipole strength computed with DH and HH, radiative decay width computed with NHH and perturbative approach) are in

good agreement when the system size is small compared to the transition wavelength of the single emitter λ_0. When the system size becomes comparable to λ_0 and the small volume approximation starts to fail, the dipole strengths computed with the DH and HH Hamiltonians are no longer good approximations to describe superradiance, but only the NHH model and the perturbative approach are still valid ($\Gamma_{SR} < \delta$, where δ is the mean energy level spacing). As already proved in Ref. S15 by some of the authors of this manuscript, if we consider larger aggregates, such as the entire chlorosome, also the perturbation approach fails, because resonances start to overlap ($\Gamma_{SR} > \delta$) and the NHH becomes the only model that can describe superradiance.

It is known that disorder and thermal noise induce localization of the excitation. For one-dimensional systems the most relevant parameter is the localization length, instead of the system length L, and the small-volume limit refers to the regime in which the localization length is smaller than λ_0, regardless of the system size L. If we assume a disorder strength that induces a localization length smaller than λ_0, for one-dimensional systems the three Hamiltonian models give always good agreement. In three-dimensional systems, however, due to the stronger influence of long-range interactions, the behavior is not so trivial. Therefore, in this case the system size has been taken as the upper bound for the localization length.

S6 General derivation of Lindblad Master Equations for independent phononic baths

S6.1 Hamiltonian and Master Equation for the whole system

Let us consider a system made of N coupled sites representing the BChl molecules in the cylinder and baseplate. All across this section for the sake of clarity we use indices i and j to indicate sites and m and n for the eigenstates, as already done in the main text. Each site is connected to an independent thermal bath. The N baths have the same temperature and the same spectral density. The full Hamiltonian is

$$\hat{H} = \hat{H}_S + \hat{H}_B + \hat{H}_I \,. \tag{S16}$$

Here $\hat{H}_S$ is the system Hamiltonian (cylinder + baseplate) already described in Sec. 3 in the main text, while the Hamiltonian term for the N independent baths is

$$\hat{H}_B = \sum_{k,i} \hbar\omega_k \hat{b}^{\dagger}_{k,i}\hat{b}_{k,i} \tag{S17}$$

where the summation runs over the modes k of the baths and the sites $i = 1, \dots, N$ and the creation/annihilation operators follow the commutation rules $[\hat{b}_{k,j}, \hat{b}^{\dagger}_{k',i}] = \delta_{k,k'}\delta_{j,i}$. Finally, the interaction is

$$\hat{H}_I = \sum_{k,i} \hbar g_k \left|i\right\rangle \left\langle i\right| \left(\hat{b}^{\dagger}_{k,i} + \hat{b}_{k,i}\right) \tag{S18}$$

where $\hbar g_k$ is the coupling between a site and the position of the k-th mode of the bath and $|i\rangle$ is a state in which the ith molecule is excited while all the others are in the ground state. Note that the derivation presented in this section does not rely on the form of $\hat{H}_S$, but only on the form of the interaction $\hat{H}_I$.

The dynamics of the full system is described by the Liouville Master Equation which, in

the interaction picture, reads

$$\frac{d\hat{\rho}(t)}{dt} = -\frac{\mathrm{i}}{\hbar}\left[\hat{H}_I(t), \hat{\rho}(t)\right] \tag{S19}$$

where $\hat{\rho}(t)$ is the density matrix in the interaction picture and it is related to the density matrix in the Schrödinger picture $\hat{\rho}$ by

$$\hat{\rho}(t) = e^{\mathrm{i}(\hat{H}_S+\hat{H}_B)t/\hbar}\,\hat{\rho}\,e^{-\mathrm{i}(\hat{H}_S+\hat{H}_B)t/\hbar}\,. \tag{S20}$$

Note that also the density matrix $\hat{\rho}$ is time-dependent both in the Schrödinger and in the interaction picture.[S16]

The interaction Hamiltonian in the interaction picture can be factorized as

$$\hat{H}_I(t) = \sum_{\omega}\sum_{i} e^{-\mathrm{i}\omega t}\hat{A}_i(\omega)\otimes\hat{B}_i(t) \tag{S21a}$$

$$= \sum_{\omega}\sum_{i} e^{\mathrm{i}\omega t}\hat{A}_i^{\dagger}(\omega)\otimes\hat{B}_i(t) \tag{S21b}$$

where $\hat{A}_i(\omega)$ are operators acting on the system,

$$\hat{A}_i(\omega) = \sum_{\substack{m,n \\ (E_n-E_m)/\hbar=\omega}} C_m^*(i)C_n(i)\,|E_m\rangle\,\langle E_n|\;, \qquad C_m(i) = \langle i|E_m\rangle \tag{S22}$$

with $|E_m\rangle$ and $|E_n\rangle$ being eigenstates of the system Hamiltonian ($\hat{H}_S\,|E_m\rangle = E_m\,|E_m\rangle$), and $\hat{B}_i(t)$ are hermitian operators acting only on the baths, given by

$$\hat{B}_i(t) = \sum_k \hbar g_k\left(\hat{b}_{k,i}^{\dagger}e^{\mathrm{i}\omega_k t} + \hat{b}_{k,i}e^{-\mathrm{i}\omega_k t}\right) \tag{S23}$$

S6.2 Born and Markov approximations

Eq. (S19) can be integrated from 0 to t to obtain

$$\hat{\rho}(t) = \hat{\rho}(0) - \frac{\mathrm{i}}{\hbar}\int_0^t dt' \left[\hat{H}_I(t'), \hat{\rho}(t')\right] \tag{S24}$$

which, substituted back into (S19), gives the integro-differential equation

$$\frac{d\hat{\rho}(t)}{dt} = -\frac{\mathrm{i}}{\hbar}\left[\hat{H}_I(t), \hat{\rho}(0)\right] - \frac{1}{\hbar^2}\int_0^t dt' \left[\hat{H}_I(t), \left[\hat{H}_I(t'), \hat{\rho}(t')\right]\right]. \tag{S25}$$

Now, let us perform the *Born approximation*: the interaction between the system and the bath is assumed to be weak, so that it does not change relevantly the status of the bath. Formally, it translates in approximating the density matrix as

$$\hat{\rho}(t) \approx \hat{\rho}_S(t) \otimes \hat{\rho}_B, \tag{S26}$$

where the bath part $\hat{\rho}_B$ is also assumed to be a steady state of the bath, i.e. $[\hat{H}_B, \hat{\rho}_B] = 0$. Making the change of variable $\tau = t - t'$ into the integral, tracing over the degrees of freedom of the bath and assuming that the average value of the positions of the bath oscillators vanish

$$\mathrm{tr}_B\left\{\left[\hat{H}_I(t), \hat{\rho}(0)\right]\right\} = 0, \tag{S27}$$

we have

$$\frac{d\hat{\rho}_S(t)}{dt} = -\frac{1}{\hbar^2}\int_0^t d\tau \,\mathrm{tr}_B\left\{\left[\hat{H}_I(t), \left[\hat{H}_I(t-\tau), \hat{\rho}_S(t-\tau) \otimes \hat{\rho}_B\right]\right]\right\}. \tag{S28}$$

Now let us perform the *Markov approximation*: the memory effects between the system and the bath are neglected, i.e. we approximate the density matrix as

$$\hat{\rho}_S(t-\tau) \approx \hat{\rho}_S(t) \tag{S29}$$

(first Markov approximation) and we extend the integration to ∞ (second Markov approximation). This gives the Redfield Master Equation

$$\frac{d\hat{\rho}_S(t)}{dt} = -\frac{1}{\hbar^2}\int_0^\infty d\tau \,\mathrm{tr}_B\left\{\left[\hat{H}_I(t), \left[\hat{H}_I(t-\tau), \hat{\rho}_S(t)\otimes\hat{\rho}_B\right]\right]\right\}. \tag{S30}$$

S6.3 Secular approximation

Let us now rewrite Eq. (S30) more explicitly. We use the notation

$$\left\langle \hat{C}\right\rangle_B = \mathrm{tr}_B\left\{\hat{C}\hat{\rho}_B\right\} \tag{S31}$$

to indicate average value of some operator $\hat{C}$ on the bath and h.c. for the Hermitian conjugate. Thus, Eq. (S30) becomes

$$\frac{d\hat{\rho}_S(t)}{dt} = \frac{1}{\hbar^2}\int_0^\infty d\tau \,\left[\left\langle \hat{H}_I(t-\tau)\hat{\rho}_S(t)\hat{H}_I(t)\right\rangle_B - \left\langle \hat{H}_I(t)\hat{H}_I(t-\tau)\hat{\rho}_S(t)\right\rangle_B\right] + \text{h.c.} \tag{S32}$$

Now we use Eq. (S21) to write the interaction Hamiltonian explicitly as

$$\hat{H}_I(t) = \sum_{\omega'}\sum_j e^{\mathrm{i}\omega' t}\hat{A}_j^\dagger(\omega')\otimes\hat{B}_j(t) \tag{S33a}$$

$$\hat{H}_I(t-\tau) = \sum_{\omega}\sum_i e^{-\mathrm{i}\omega(t-\tau)}\hat{A}_i(\omega)\otimes\hat{B}_i(t-\tau) \tag{S33b}$$

and, substituting into (S32), we have

$$\frac{d\hat{\rho}_S(t)}{dt} = \sum_{\omega,\omega'} e^{\mathrm{i}(\omega'-\omega)t}\sum_{j,i}\Gamma_{ji}(\omega,t)\left[\hat{A}_i(\omega)\hat{\rho}_S(t)\hat{A}_j^\dagger(\omega') - \hat{A}_j^\dagger(\omega')\hat{A}_i(\omega)\hat{\rho}_S(t)\right] + \text{h.c.} \tag{S34}$$

where we have defined the rates $\Gamma_{ji}(\omega,t)$ as the half-sided Fourier transformed correlators of the bath

$$\Gamma_{ji}(\omega,t) = \frac{1}{\hbar^2}\int_0^\infty d\tau\, e^{\mathrm{i}\omega\tau}\left\langle \hat{B}_j(t)\hat{B}_i(t-\tau)\right\rangle_B. \tag{S35}$$

Since we assumed $\hat{\rho}_B$ stationary, the rates $\Gamma_{ji}(\omega)$ are independent of time, namely

$$\Gamma_{ji}(\omega) = \frac{1}{\hbar^2} \int_0^\infty d\tau \, e^{\mathrm{i}\omega\tau} \left\langle \hat{B}_j(\tau)\hat{B}_i(0) \right\rangle_B . \tag{S36}$$

Now let us perform the *secular approximation*: we neglect the oscillating terms, assuming that the energy spacings between the system states induces Rabi oscillations which are faster than the relaxation time so that, in a coarse-grained time scale, these oscillations average to 0. So, keeping just the non-oscillating terms ($\omega = \omega'$), we get a Master Equation in the Lindblad form:

$$\frac{d\hat{\rho}_S(t)}{dt} = \sum_{\omega} \sum_{j,i} \Gamma_{ji}(\omega) \left[\hat{A}_i(\omega)\hat{\rho}_S(t)\hat{A}_j^\dagger(\omega) \; - \hat{A}_j^\dagger(\omega)\hat{A}_i(\omega)\hat{\rho}_S(t) \right] + \text{h.c.} \tag{S37}$$

It is important to stress that the positivity of populations is guaranteed only by the secular approximation. Redfield Eq. (S30) can have solutions with negative populations.

S6.4 Explicit calculation of the rates

Here we compute the rates $\Gamma_{ji}(\omega)$ for the specific case of identical independent thermal baths under consideration. Note that these rates appear both in the Redfield Master Equation (S34) and in the Lindblad Master Equation (S37). By substituting the expressions (S23) into (S36) we have

$$\begin{aligned} \Gamma_{ji}(\omega) = \int_0^\infty d\tau \, e^{\mathrm{i}\omega\tau} \sum_{k,j,k',i} g_k g_{k'} \Big[& e^{-\mathrm{i}\omega_k\tau} \left\langle \hat{b}_{k,j}\hat{b}_{k',i} \right\rangle_B + e^{-\mathrm{i}\omega_k\tau} \left\langle \hat{b}_{k,j}\hat{b}_{k',i}^\dagger \right\rangle_B \\ & + e^{\mathrm{i}\omega_k\tau} \left\langle \hat{b}_{k,j}^\dagger\hat{b}_{k',i} \right\rangle_B + e^{\mathrm{i}\omega_k\tau} \left\langle \hat{b}_{k,j}^\dagger\hat{b}_{k',i}^\dagger \right\rangle_B \Big] . \end{aligned} \tag{S38}$$

Now we assume that the thermal baths are at thermal equilibrium, i.e.

$$\hat{\rho}_B = \frac{e^{-\beta \hat{H}_B}}{\mathrm{tr}_B\left\{e^{-\beta \hat{H}_B}\right\}} \tag{S39}$$

where $\beta = 1/(k_B T)$ is the inverse temperature. In this case one can show that the correlators in (S38) are

$$\left\langle \hat{b}_{k,j}\hat{b}_{k',i}\right\rangle_B = 0 \tag{S40a}$$

$$\left\langle \hat{b}^\dagger_{k,j}\hat{b}^\dagger_{k',i}\right\rangle_B = 0 \tag{S40b}$$

$$\left\langle \hat{b}_{k,j}\hat{b}^\dagger_{k',i}\right\rangle_B = \delta_{k,k'}\delta_{j,i}\left(1 + N_{BE}(\omega_k)\right) \tag{S40c}$$

$$\left\langle \hat{b}^\dagger_{k,m}\hat{b}_{k',n}\right\rangle_B = \delta_{k,k'}\delta_{j,i} N_{BE}(\omega_k) \tag{S40d}$$

where we have defined the Bose-Einstein function

$$N_{BE}(\omega_k) = \frac{1}{e^{\beta\hbar\omega_k} - 1}\,. \tag{S41}$$

Thus we have

$$\Gamma_{ji}(\omega) = \delta_{j,i}\int_0^\infty d\tau\, e^{\mathrm{i}\omega\tau}\sum_k g_k^2\left[e^{-\mathrm{i}\omega_k\tau}\left(1 + N_{BE}(\omega_k)\right) + e^{\mathrm{i}\omega_k\tau}N_{BE}(\omega_k)\right]. \tag{S42}$$

As regards the sum over k, we take the continuum limit

$$\sum_k g_k^2 f(\omega_k) \to \int_0^\infty d\omega_k\, J(\omega_k) f(\omega_k)\,, \tag{S43}$$

where $J(\omega_k)$ is the spectral density and $f(\omega_k)$ is the function in the squared brackets.

Now we perform the integral over τ using the relation

$$\int_0^\infty d\tau\, e^{\mathrm{i}\omega\tau} = \pi\delta(\omega) + \mathrm{i}\mathrm{P}\frac{1}{\omega} \tag{S44}$$

where P is the Cauchy principal value. So, we can split the rates into their real and an imaginary parts,

$$\Gamma_{ji}(\omega) = \frac{1}{2}\gamma_{ji}(\omega) + \mathrm{i}S_{ji}(\omega) \tag{S45}$$

which are, respectively,

$$\gamma_{ji}(\omega) =2\pi\delta_{j,i}\int_0^\infty d\omega_k\, J(\omega_k)\left[\delta(\omega-\omega_k)\left(1+N_{BE}(\omega_k)\right)+\delta(\omega+\omega_k)N_{BE}(\omega_k)\right] \tag{S46a}$$

$$S_{ji}(\omega) =\delta_{j,i}\mathrm{P}\int_0^\infty d\omega_k\, J(\omega_k)\left[\frac{1+N_{BE}(\omega_k)}{\omega-\omega_k}+\frac{N_{BE}(\omega_k)}{\omega+\omega_k}\right] \tag{S46b}$$

On integrating the $\delta(\omega \pm \omega_k)$ functions we have the real part of the rates

$$\gamma_{ji}(\omega) = 2\pi\delta_{j,i}\left[J(\omega)\left(1+N_{BE}(\omega)\right)+J(-\omega)N_{BE}(-\omega)\right] = \gamma^{(p)}(\omega)\delta_{j,i}\,, \tag{S47}$$

where we have defined the diagonal rate $\gamma^{(P)}(\omega)$ implicitly. The imaginary parts $S_{mn}(\omega)$ of the rates induce a constant renormalization of the site energies and thus they can be neglected.

S6.5 Lindblad Master Equations on the system eigenbasis

The Lindblad Master Equations may be written back to the Schrödinger picture as

$$\frac{d\hat{\rho}_S}{dt} = -\frac{\mathrm{i}}{\hbar}\left[\hat{H}_S, \hat{\rho}_S\right] + \mathcal{L}_T[\hat{\rho}_S] \tag{S48}$$

where the dissipator $\mathcal{D}_L[\hat{\rho}_S]$ in the Lindblad case (S34) is

$$\mathcal{L}_T[\hat{\rho}_S] = \sum_\omega \gamma^{(p)}(\omega)\sum_i\left[\hat{A}_i(\omega)\hat{\rho}_S\hat{A}_i^\dagger(\omega) - \frac{1}{2}\left\{\hat{A}_i^\dagger(\omega)\hat{A}_i(\omega), \hat{\rho}_S\right\}\right]\,. \tag{S49}$$

We now proceed to write the Master Equation on the eigenbasis of $\hat{H}_S$, i.e. we use the expression (S22) to compute the terms $\langle E_m|\,\mathcal{L}_T\,|E_n\rangle$.

For simplicity, let us assume that the coefficients $C_m(i) = \langle i|E_m\rangle$ are all real numbers.

Since $\hat{H}_S$ is hermitian, it is always possible to find an eigenbasis that satisfies this requirement.

The dynamics of the populations is given by

$$\langle E_m| \mathcal{L}_T |E_m\rangle = \sum_n (T_{mn}\rho_{nn} - T_{nm}\rho_{mm}) \tag{S50}$$

while for the coherences ($m \neq n$) we have

$$\langle E_m| \mathcal{L}_T |E_n\rangle = -\Gamma_{mn}\rho_{mn} \tag{S51}$$

and

$$\langle 0| \mathcal{L}_T |E_m\rangle = -\Gamma_{0m}\rho_{0m}\,. \tag{S52}$$

Defining the coefficients

$$\Lambda_{mn} = \sum_i |C_m(i)|^2 |C_n(i)|^2\,, \tag{S53}$$

we derive the relaxation rates for the populations, expressed as

$$T_{mn} = \gamma^{(p)}[(E_n - E_m)/\hbar]\Lambda_{mn}. \tag{S54}$$

The Lindblad Master Equation for the Hamiltonian eigenstate populations reads explicitly:

$$\begin{aligned}\frac{d\rho_{mm}}{dt} = &\sum_n \{2\pi\left[J(\omega_n - \omega_m)\left(1 + N(\omega_n - \omega_m)\right) + J(\omega_m - \omega_n)N(\omega_m - \omega_n)\right]\Lambda_{mn}\rho_{nn} \\ &-2\pi\left[J(\omega_m - \omega_n)\left(1 + N(\omega_m - \omega_n)\right) + J(\omega_n - \omega_m)N(\omega_n - \omega_m)\right]\Lambda_{mn}\rho_{nn}\}\ ,\end{aligned} \tag{S55}$$

where we chose $J(\omega) = J(\omega_n - \omega_m) = k_{vib}\omega$ as the phonon spectral density already used by some of the authors of this manuscript in S3.

S7 Multichromophoric transfer rates MC-FRET

In this section a deeper study of the MC-FRET is given. MC-FRET has already been used in literature to model the incoherent energy transfer between different aggregates[S17–S19] and it is computed by using the Förster rate K_{nm} defined in Eq. (22) in the main text. K_{nm} depends mainly on two quantities: the overlap between the emission spectrum of the donor and the absorption spectrum of the acceptor, and the coupling strength between the eigenstates of the two aggregates. The former depends on the parameter Γ_ϕ.

An aggregate absorption spectrum is given by:[S20–S22]

$$A(E) \propto \sum_n \mu^2 |D_n|^2 A_n(E), \tag{S56}$$

where $|D_n|^2$ and $A_n(E)$ are, respectively, the dipole strength and the normalized lineshape for each n eigenstate.

The emission spectrum, on the other hand, is

$$F(E) \propto \sum_m \mu^2 |D_m|^2 F_m(E), \tag{S57}$$

where the emission lineshapes are multiplied by the thermal populations p_m, see Eq. (29) in the main text, namely

$$F_m(E) = p_m A_m(E). \tag{S58}$$

For high temperature and short bath correlation time,[S17,S22] we can neglect the phonon-induced Stokes and anti-Stokes shifts and approximate all the absorption lines as Lorentzians:

$$A_n(E) = \frac{2\Gamma_\phi}{\Gamma_\phi^2 + (E - E_n)^2} \tag{S59}$$

peaked at the eigenstate energy E_n and with a dephasing-induced linewidth Γ_ϕ. Note that the normalization condition gives $\int_{-\infty}^{+\infty} A_n(E) dE = 2\pi$.

Fig. S5 shows the overlap between the emission and absorption spectra of MT, PD and RD cylinders and baseplate respectively, assuming a Lorentzian linewidth of 500 cm^{-1} for both cylinders and baseplate spectra. The choice of the linewidth for the emission and absorption spectra is consistent with experimental data. See Ref. S15 for a more detailed comparison between experimental and numerical spectra in GSB antenna complexes, where a similar linewidth has been used.

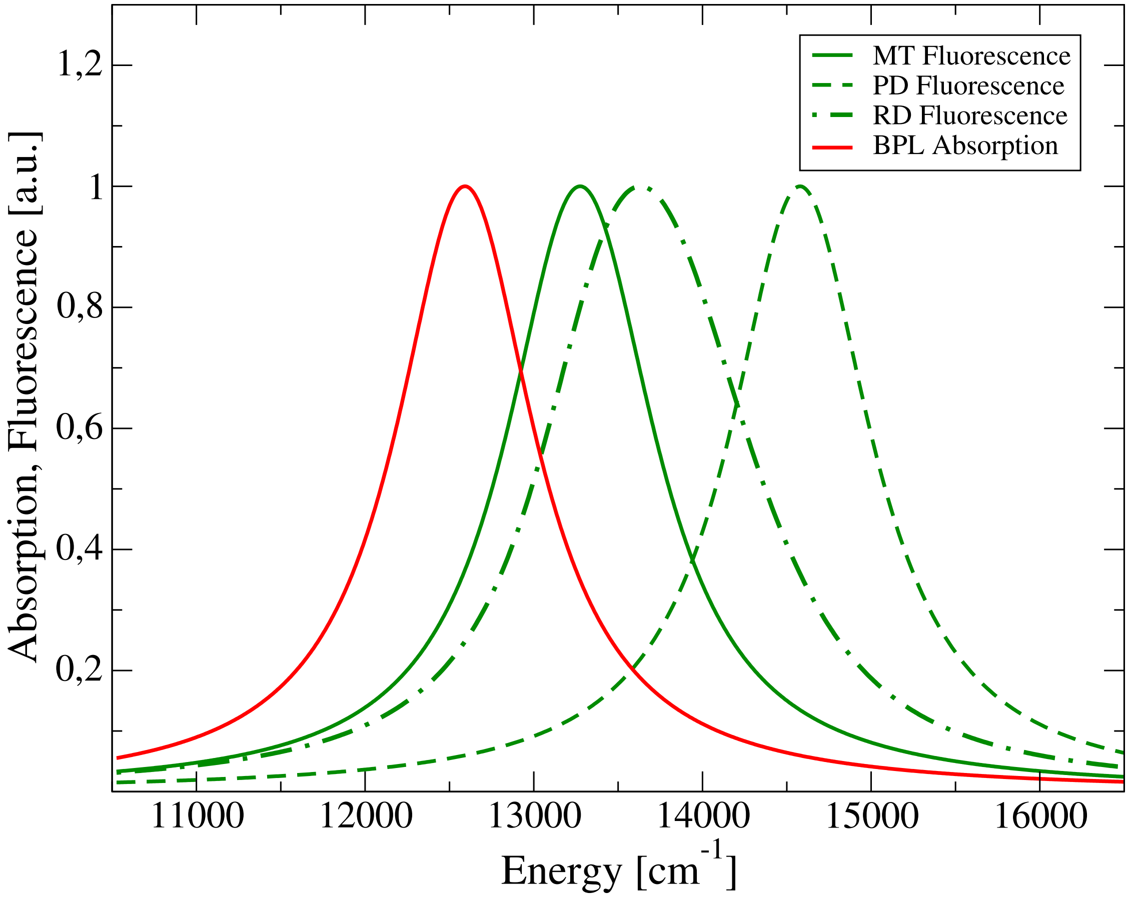

Figure S5: *Emission and absorption spectra in MT, PD, RD cylinders and baseplate.* Normalized emission spectra for the donor aggregates in green color, see Eq. (S57). Continuous line for the MT model, dashed line for the PD model and dashed-dotted line for the RD model. The red continuous line represents the absorption spectrum of the baseplate, see Eq. (S56). Here a Lorentzian lineshape with linewidth of 500 cm^{-1} has been considered for all the models.

Furthermore the couplings $\Omega_{n,m}$ between cylinder and baseplate eigenstates are studied. If $\hat{H}_{DH}$ is the Hamiltonian of the entire system (cylinder + baseplate) written on the site

basis, see Sec. 3 in the main text, the coupling strength between the mth eigenstate of the cylinder $|E_m\rangle$ and the nth eigenstate of the baseplate $|E_n\rangle$ reads:

$$\Omega_{n,m} = \langle E_m| \hat{H}_{DH} |E_n\rangle = \sum_{i \in C, j \in B} C_m^*(i) C_n(j) H_{DH}(i,j), \tag{S60}$$

where $C_m(i)$ and $C_n(j)$ are the projections of the eigenstates on the sites basis, while $H_{DH}(i,j)$ is the coupling computed with the Frenkel Hamiltonian between sites i and j belonging respectively to the cylinder and baseplate.

Finally, the following calculations justify the choice of the parameter $\Gamma_\phi = 1000$ cm^{-1}, where $\Gamma_\phi/\hbar$ is the dephasing rate, in Eq. (22) in the main text as the sum of the absorption and emission spectra linewidths.

The MC-FRET rate is usually expressed as

$$K_{D,A} = \sum_{m \in D} \sum_{n \in A} \frac{|\Omega_{n,m}|^2}{2\pi\hbar} \int_{-\infty}^{+\infty} F_m(E) A_n(E) dE = \sum_{m \in D} \sum_{n \in A} p_m \, K_{n,m}, \tag{S61}$$

where $\Omega_{n,m} = \langle E_m|H_{DH}|E_n\rangle$ is the Hamiltonian matrix element between the m donor eigenstate and the m acceptor eigenstate computed in Eq. (S60).

Under the assumption made before for the absorption and emission lineshapes, the overlap integral in equation (S61) is analytically computed. If Γ_d and Γ_a are the linewidths for the donor and acceptor aggregates respectively, the overlap integral reads

$$\int_{-\infty}^{+\infty} F_m(E) A_n(E) dE = p_m \int_{-\infty}^{+\infty} \frac{4\Gamma_d \Gamma_a}{[\Gamma_d^2 + (E - E_m)^2][\Gamma_a^2 + (E - E_n)^2]} dE. \tag{S62}$$

The integral in Eq. (S62) can be solved using Jordan's lemma and the final expression is as follows:

$$\int_{-\infty}^{+\infty} F_m(E) A_n(E) dE = p_m \frac{4\pi(\Gamma_d + \Gamma_a)}{(\Gamma_d + \Gamma_a)^2 + (E_m - E_n)^2}. \tag{S63}$$

Eq. (S63) shows that the overlap integral of the two Lorentzian functions with Γ_d and Γ_a

linewidths is still a Lorentzian function peaked at $E_n - E_m$ and with a total dephasing-induced linewidth which is the sum of Γ_d and Γ_a.

Therefore, if $\Gamma_\phi = \Gamma_a + \Gamma_d$, we can express the MC-FRET rate in equation (S61) as

$$K_{D,A} = \sum_{m\in D}\sum_{n\in A} p_m K_{n,m}, \tag{S64}$$

where the transfer rates between a m donor eigenstate and a n acceptor eigenstate are

$$K_{n,m} = \frac{|\langle E_m|H_{DH}|E_n\rangle|^2}{\hbar}\cdot\frac{2\Gamma_\phi}{\Gamma_\phi^2 + (E_m - E_n)^2}. \tag{S65}$$

Note that these rates are symmetric, $K_{n,m} = K_{m,n}$, causing Eq. (S65) to break detailed balance. Since in the GSB aggregates that we consider here usually the donor and acceptor eigenvalues are not resonant, we correct these rates by using Eq. (S65) only for energetically downward transitions, otherwise taking $K_{n,m} = K_{m,n}e^{-(E_n-E_m)/k_BT}$. This method has already been used in Refs. S13,S23 in order to describe excitation energy transfer in Purple bacteria light-harvesting aggregates. In the main text (see Eq. (22)) the transfer rates $K_{n,m}$ have been given by adding this correction. As a consequence, if both donor and acceptor aggregates are made of one molecule, the MC-FRET gives forward and backward rates which are detailed balance.

S8 Parameters of the model

In this section a brief discussion of the main parameters adopted in our simulations has been provided in order to give a validation of the model under consideration.

- We kept the intrinsic radiative decay rate of each molecule constant and we determined the inter-molecular couplings according to the radiative non-hermitian Hamiltonian shown Eq. (1) in the main text, strictly dependent on the geometry of the system. The consistency of our modeling has been verified by reproducing experimental spectroscopic data of absorption and emission spectra for GSB light-harvesting complexes, see Fig. S6 and our previous work S15.

- The dephasing strength Γ_Φ used to determine the FRET rates between eigenstates belonging to different aggregates was tuned to match the linewidth of the experimental emission and absorption spectra. However, a 30% variation of Γ_Φ has been considered in order to assess the parameter's sensitivity. This is shown in Fig. S7, where the trapped current per RC (panel A) and the internal efficiency (panel B) as a function of Γ_Φ are represented for a cylinder (MT model) with 6000 Bchl *c* coupled to a baseplate with 2184 Bchl *a* molecules. The foundings shown in Fig. S7 result in only a small discrepancy compared to the value used to fit the spectra ($\Gamma_\Phi = 1000$ cm^{-1}, which represents the sum of the absorption and emission linewidths).

- Finally, the non-radiative decay rate has been kept constant, adopting a standard value consistent with existing literature for these systems.[S23] While the parameter k_{vib} in the spectral density $J(\omega)$ has been chosen in order to ensure thermal relaxation within each aggregate in a few picoseconds.

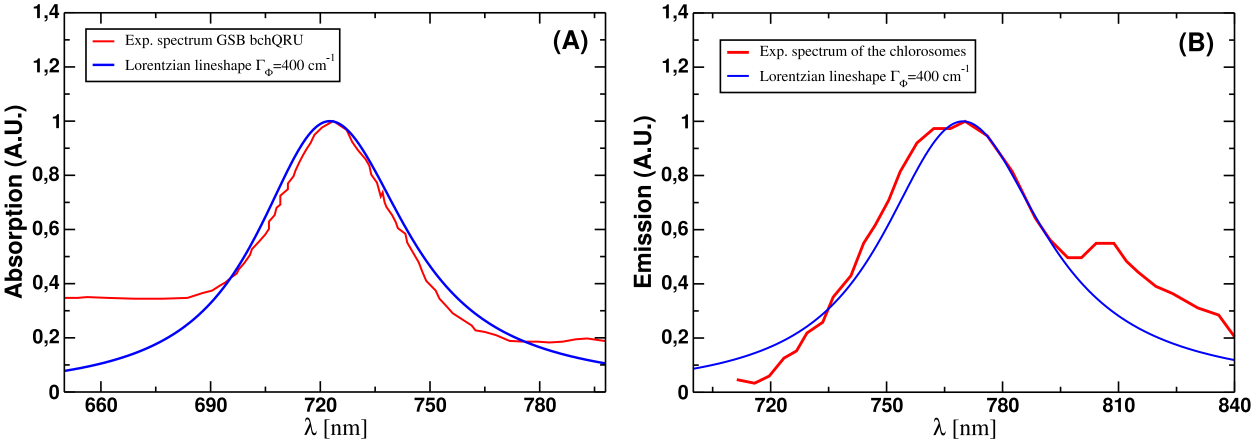

Figure S6: *Absorption and emission spectra in GSB.* The figure shows the comparison between the experimental and numerical absorption and emission spectra for the GSB model, see panels (A) and (B), respectively. Panel (A): experimental and numerical normalized absorption spectra for GSB bchQRU triple mutant are compared. The experimental absorption spectrum of whole cells of GSB bchQRU mutant (red solid line) taken from Ref. S24 is compared with the numerical spectrum for a single cylinder with 180 rings (blue solid line), assuming a Lorentzian lineshape with homogeneous broadening $\Gamma_\Phi = 400$ cm^{-1}. Panel (B): experimental and numerical normalized emission spectra for GSB are compared. The experimental emission spectrum of chlorosomes of GSB (red solid line) taken from Ref. S10 is compared with the numerical spectrum for a single cylinder with 180 rings (blue solid line), assuming a Lorentzian lineshape with homogeneous broadening $\Gamma_\Phi = 400$ cm^{-1} and room temperature.

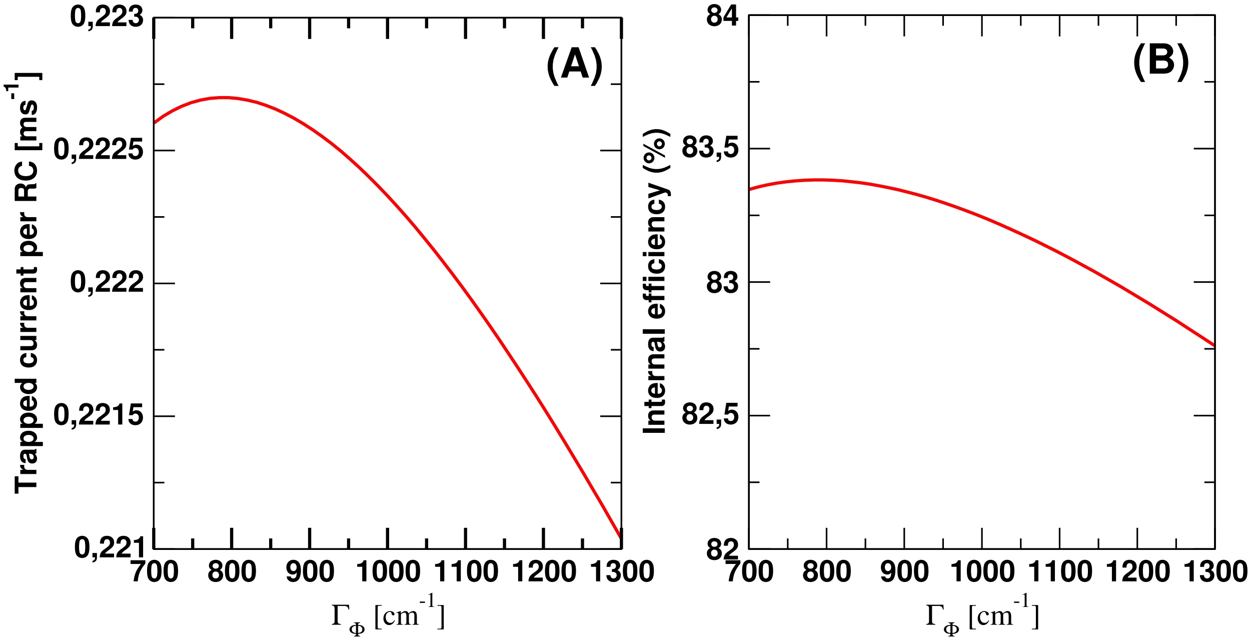

Figure S7: *Trapped current and efficiency in MT model vs dephasing linewidth* Γ_Φ. The figure shows the trapped current (panel A) and the internal efficiency (panel B) as a function of the dephasing linewidth Γ_Φ for a cylinder (MT model) with 6000 Bchl *c* coupled to a baseplate with 2184 Bchl *a* molecules.

S9 Size-dependent energy transfer efficiency and trapped current in GSB light-harvesting architectures

In this section a deeper study of the energy transfer efficiency and trapped current dependence on the system size is addressed. In our calculations we investigated two different geometries in order to describe natural models: a full-scale system comprising more than 10^5 Bchl *c* molecules arranged on three adjacent concentric cylinders, that mimics the natural light-harvesting architectures, and a smaller model consisting of a single cylindrical aggregate of 6000 BChl *c* molecules (MT model). Our results provided in the main text (Figs. 6 and 7, panel B) demonstrate that both models achieve internal efficiencies between 70% and 85%, consistent with literature values. However, the single cylinder model has a much smaller trapped current which fails to match the RC closure rate. Indeed, the single-cylinder system yields a trapped current an order of magnitude lower, resulting in sub-optimal operating conditions. Only the full chlorosome model generates a trapped current comparable to the RC closure rate, thereby optimizing the energy transfer process (Fig. 7, panel A). These findings are summarized in Fig. S8, where the trapped current and the internal efficiency have been shown for the entire chlorosome and for a single cylinder (MT) with two different dimensions coupled to a baseplate.

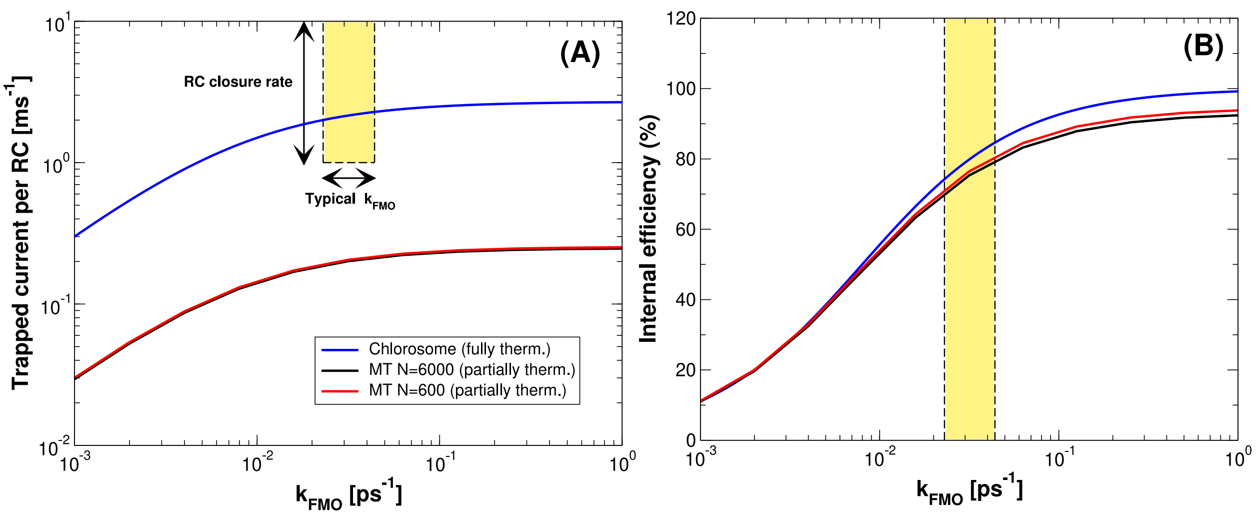

Figure S8: *Study of the size-dependence of the energy transfer and trapped current in GSB light-harvesting complexes.* The figure shows the trapped current per RC (panel A) and the internal efficiency (panel B) as a function of the k_{FMO} trapping rate. The yellow windows represent the regime where GSB typically work. In both panels the comparison between the entire chlorosome (blue line) and a single cylindrical MT model coupled to a baseplate has been represented. For the single MT cylinder two different sizes have been considered: the one already studied in the manuscript containing N=6000 Bchl c (black line) and a smaller one where N=600 Bchl c molecules are arranged on a cylinder made of 10 rings (red line). For the entire chlorosome the full rate equations approach has been used to compute both trapped current and internal efficiency, while for the single MT cylinders the partially thermalized model has been employed.

S10 Analysis of TDMs distribution in a cone

In the main text we clearly demonstrate, through explicit comparisons between the MT, PD and RD models, that light-harvesting performance is strongly influenced by the orientation of transition dipole moments, with the natural geometry yielding the highest trapped current and internal efficiency. In this section we provide a further study in order to investigate and identify a tolerance range for the β angle. A MT cylinder with 6000 Bchl c coupled to a baseplate with 2184 Bchl a molecules has been considered. In this model the β angle is no longer kept constant, but randomly and uniformly distributed in a cone around the main direction of the original TDMs. Fig. S9 shows how the solid angle cone aperture (δ) (from zero to 4π) affects both the internal efficiency and the trapped current. For each δ angle the average values of trapped current and internal efficiency have been determined over 10 realizations. The obtained results show that both internal efficiency and trapped current start to drop as the cone aperture increases, reaching the RD model for $\delta > 2\pi$ (see the area between the two black dashed lines which represents the range values obtained with the RD model). Specifically the trapped current is decreased by 4% and the efficiency by the 1.6% for $\delta = \pi(2 - \sqrt{2})$. These findings suggest that while the system is optimized for the β close to 55°, it maintains a degree of robustness against moderate angular fluctuations.

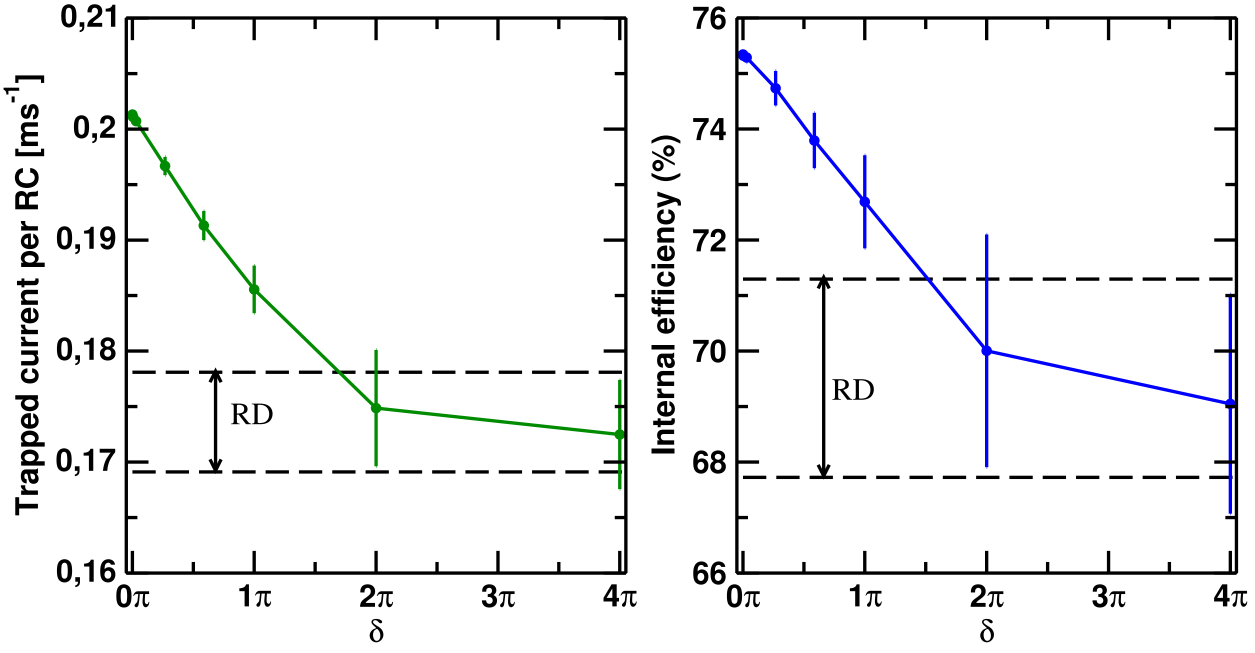

Figure S9: *Analysis of trapped current and internal efficiency assuming a TDMs distribution in a cone.* The figure shows the trapped current (panel A) and the internal efficiency (panel B) as a function of the cone aperture δ for a system comprising a single cylinder (MT model) with 6000 Bchl c coupled to a baseplate with 2184 Bchl a molecules. The cone aperture is defined by the solid angle $0 \leq \delta \leq 4\pi$. Specifically, the case $\delta = 0$ corresponds to the MT model, where all the TDMs form an angle $\beta = 55°$ with respect to the cylinder axis. Conversely, $\delta = 4\pi$ represents a uniform random distribution within a sphere and it corresponds to the RD model. For each δ angle the average values of trapped current and internal efficiency have been determined over 10 realizations. The two dashed black lines in both panel A and B represent the range values obtained with the RD model.

S11 Comparison between MT and WT models

GSB light-harvesting systems studied in the main text belong to the family of the mutant type model, obtained by genetic modifications of the system found in nature, which is the wild type model (WT). Experimental studies reveal that the WT chlorosomes are much more heterogeneous than chlorosomes from the MT model, furthermore the half-band width of the Q_y absorption maximum of the BChl c aggregates is largest for WT chlorosomes isolated from cells grown at low light intensity and it is also much broader than for chlorosomes of the MT model.[S25,S26]

In this section we propose a comparison between the mutant type (MT) and the wild type (WT) models for the GSB light-harvesting single-wall nanotubes. In this manuscript the geometry of the WT and MT models has been determined starting from the 2-dimensional Bravais lattice and wrapping it up according to two different rolling vectors, which are mutually perpendicular, see Ref. S26 for a description of the Bravais lattice. The result is that in the MT model BChls are organized into equal, horizontal and coaxial rings, while in the WT model BChls are organized into vertical chains, originating a helical structure. A brief explanation of the geometry of the WT model is provided in Fig. S10, while a wider explanation of the geometry of all the single-walled models can be found in Ref. S5. Here we compare the trapped current and internal efficiency for both MT and WT models, computed by using the partially thermalized rate equations approach described in Sec. 4.2 in the main text. Fig. S11 shows the trapped current (panel A) and the internal efficiency (panel B) as a function of the FMO trapping rate. Our foundings demonstrate that MT and WT models have a similar behavior: both of them show a trapped current between $1 - 3 \times 10^{-1}$ ms^{-1} and an internal efficiency between $70 - 80\%$, see the yellow box that represents the typical FMO trapping rate range. These results confirm that the realistic models (both WT and MT) exploit excitation energy transfer efficiently and they are able to funnel almost all the absorbed excitation to the RCs with higher performance than the other mathematical models.

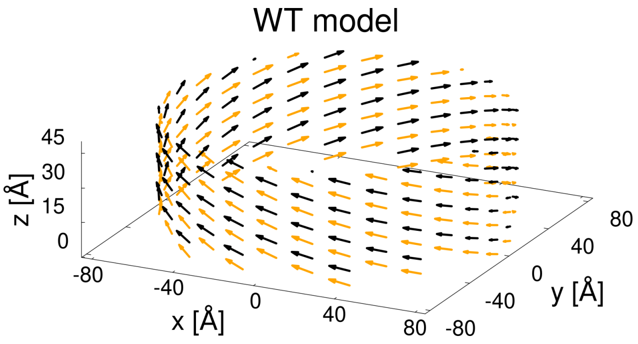

Figure S10: *Geometry of the wild type (WT) model.* The figure represents a section of the wild type antenna complex. Positions and orientations of the TDMs associated to each BChl c molecule are represented by orange and black arrows. For the sake of clarity we show only 30 dipoles per ring instead of 60 as we considered in this paper. Moreover the distances along the z-axis are enhanced by a factor of 5 with respect to the distances on the x–y axes. The WT model can be thought as organized into vertical chains to originate a helical structure. Also in the WT model there is the alternation $\pm 4°$ between consecutive dipoles on the same chain, here represented by the alternation between black and orange arrows. For more details about the structure of the WT model see Ref. S5.

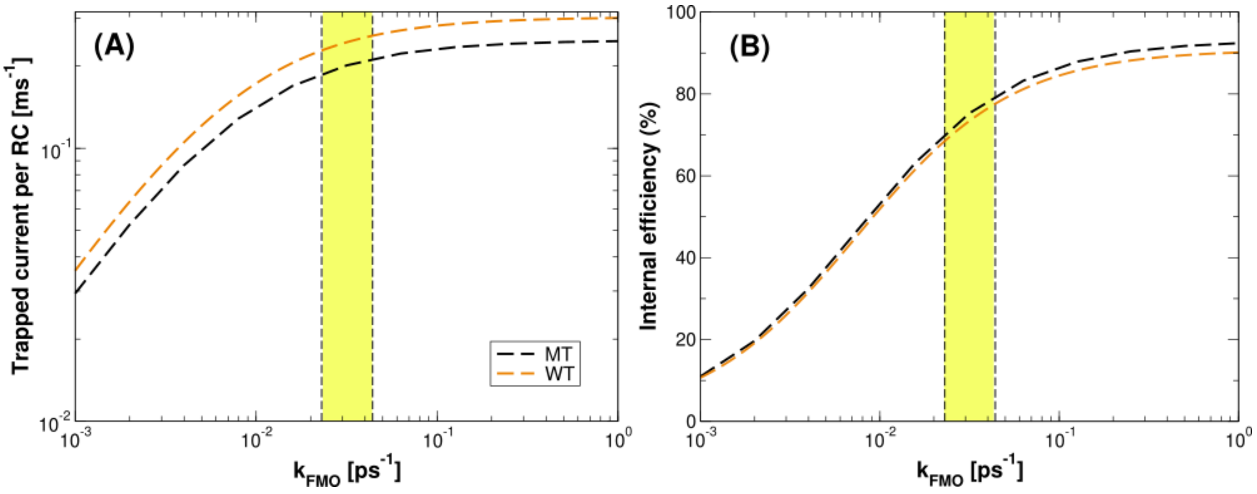

Figure S11: *Comparison between WT and MT models.* Trapped current (panel A) and internal efficiency (panel B) as a function of the k_{FMO} trapping rate for MT and WT models coupled to a dimeric baseplate. The results have been obtained with the partially thermalized model using Eqs. (30) and (31) for the trapped current and the internal efficiency, respectively. For the MT model we consider a cylinder made of 6000 BChl c molecules and a dimeric baseplate containing 2184 BChl a molecules. For the WT model a cylinder with 6000 BChl c molecules and a dimeric baseplate containing 1608 BChl a molecules have been considered. More details about the size of the aggregates and the number of BChl molecules are provided in Tab. S3. The yellow window between the two dashed lines represent the region where the k_{FMO} trapping rate typically works in GSB species.

Supporting References

(S1) Olšina, J.; Dijkstra, A. G.; Wang, C.; Cao, J. Can natural sunlight induce coherent exciton dynamics? *arXiv preprint* **2014**, arXiv:1408.5385.

(S2) Würfel, P.; Würfel, U. *Physics of solar cells: from basic principles to advanced concepts*; John Wiley & Sons, 2016.

(S3) Mattiotti, F.; Brown, W. M.; Piovella, N.; Olivares, S.; Gauger, E. M.; Celardo, G. L. Bio-inspired natural sunlight-pumped lasers. *New Journal of Physics* **2021**, *23*, 103015.

(S4) Let us recall that, in Gaussian units, the unit dipole-dipole interaction energy is $[E] = [\mu]^2[d]^{-3}$, where $[\mu]$ is the unit dipole and $[d]$ the unit distance. We express the dipoles in D (Debye), the distance in Å and the energy in cm^{-1} units (applying the standard conversion $[E]/(hc)$, with h being the Planck constant and c the speed of light), so that $[\mu]^2/(hc) = \mathrm{cm}^{-1}\mathrm{\AA}^3$. Now, from the definition $1\ \mathrm{D} = 10^{-18}\ \mathrm{cm}^{5/2}\ \mathrm{g}^{1/2}\ \mathrm{s}^{-1}$ we have $1\ \mathrm{D}^2 = 10^{-12}\ \mathrm{cm}^2\ \mathrm{g}\ \mathrm{s}^{-2}\ \mathrm{\AA}^3$. Recalling the Planck constant $h = 6.626 \cdot 10^{-27}\ \mathrm{cm}^2\ \mathrm{g}\ \mathrm{s}^{-1}$ and the speed of light $c = 2.998 \cdot 10^{10}\ \mathrm{cm}\ \mathrm{s}^{-1}$, we have $1\ \mathrm{D}^2/(hc) = 5034\ \mathrm{cm}^{-1}\ \mathrm{\AA}^3$. So, a transition dipole $\mu = \sqrt{30}\ \mathrm{D}$ results in $|\mu|^2 = 30 \times 5034\ \mathrm{cm}^{-1}\ \mathrm{\AA}^3 = 151\,020\ \mathrm{cm}^{-1}\ \mathrm{\AA}^3$. Note that in these calculations we write explicitly where the energy is divided by hc for clarity, while in the main text we always assume implicitly that any energy is divided by hc.

(S5) Gullí, M.; Valzelli, A.; Mattiotti, F.; Angeli, M.; Borgonovi, F.; Celardo, G. L. Macroscopic coherence as an emergent property in molecular nanotubes. *New J. Phys.* **2019**, *21*, 013019.

(S6) Linnanto, J. M.; Korppi-Tommola, J. E. I. Exciton Description of Chlorosome to Baseplate Excitation Energy Transfer in Filamentous Anoxygenic Phototrophs and Green Sulfur Bacteria. *J. Phys. Chem. B* **2013**, *117*, 11144–11161.

(S7) Connolly, J. S.; Samuel, E. B.; Janzen, A. F. Effects of solvent on the fluorescence properties of bacteriochlorophyll a. *Photochemistry and photobiology* **1982**, *36*, 565–574.

(S8) Blankenship, R. E. *Molecular mechanisms of photosynthesis*; John Wiley & Sons, 2021.

(S9) Borrego, C. M.; Arellano, J. B.; Abella, C. A.; Gillbro, T.; Garcia-Gil, J. The molar extinction coefficient of bacteriochlorophyll e and the pigment stoichiometry in Chlorobium phaeobacteroides. *Photosynthesis research* **1999**, *60*, 257–264.

(S10) Malina, T.; Koehorst, R.; Bína, D.; Pšenčík, J.; van Amerongen, H. Superradiance of bacteriochlorophyll c aggregates in chlorosomes of green photosynthetic bacteria. *Scientific reports* **2021**, *11*, 8354.

(S11) Pšenčík, J.; Butcher, S. J.; Tuma, R. Chlorosomes: structure, function and assembly. *The structural basis of biological energy generation* **2014**, 77–109.

(S12) Stanier, R.; Smith, J. The chlorophylls of green bacteria. *Biochimica et biophysica acta* **1960**, *41*, 478–484.

(S13) Baghbanzadeh, S.; Kassal, I. Distinguishing the roles of energy funnelling and delocalization in photosynthetic light harvesting. *Phys. Chem. Chem. Phys.* **2016**, *18*, 7459–7467.

(S14) Huh, J.; Saikin, S. K.; Brookes, J. C.; Valleau, S.; Fujita, T.; Aspuru-Guzik, A. Atomistic Study of Energy Funneling in the Light-Harvesting Complex of Green Sulfur Bacteria. *J. Am. Chem. Soc.* **2014**, *136*, 2048–2057.

(S15) Valzelli, A.; Boschetti, A.; Mattiotti, F.; Kargol, A.; Green, C.; Borgonovi, F.; Celardo, G. L. Large scale simulations of photosynthetic antenna systems: interplay

of cooperativity and disorder. *The Journal of Physical Chemistry B* **2024**, *128*, 9643–9655.

(S16) Breuer, H.-P.; Petruccione, F. *The theory of open quantum systems*; OUP Oxford, 2002.

(S17) Cleary, L.; Cao, J. Optimal thermal bath for robust excitation energy transfer in disordered light-harvesting complex 2 of purple bacteria. *New J. Phys.* **2013**, *15*, 125030.

(S18) Ma, J.; Cao, J. Förster resonance energy transfer, absorption and emission spectra in multichromophoric systems. I. Full cumulant expansions and system-bath entanglement. *J. Chem. Phys.* **2015**, *142*, 094106.

(S19) Strümpfer, J.; Sener, M.; Schulten, K. How quantum coherence assists photosynthetic light-harvesting. *The journal of physical chemistry letters* **2012**, *3*, 536–542.

(S20) Renger, T.; Marcus, R. A. On the relation of protein dynamics and exciton relaxation in pigment–protein complexes: An estimation of the spectral density and a theory for the calculation of optical spectra. *The Journal of chemical physics* **2002**, *116*, 9997–10019.

(S21) Babcock, N.; Montes-Cabrera, G.; Oberhofer, K.; Chergui, M.; Celardo, G.; Kurian, P. Ultraviolet superradiance from mega-networks of tryptophan in biological architectures. *The Journal of Physical Chemistry B* **2024**,

(S22) Mattiotti, F.; Sarovar, M.; Giusteri, G. G.; Borgonovi, F.; Celardo, G. L. Efficient light harvesting and photon sensing via engineered cooperative effects. *New Journal of Physics* **2022**, *24*, 013027.

(S23) Baghbanzadeh, S.; Kassal, I. Geometry, supertransfer, and optimality in the light

harvesting of purple bacteria. *The Journal of Physical Chemistry Letters* **2016**, *7*, 3804–3811.

(S24) Chew, A. G. M.; Frigaard, N.-U.; Bryant, D. A. Bacteriochlorophyllide c C-82 and C-121 methyltransferases are essential for adaptation to low light in Chlorobaculum tepidum. *Journal of bacteriology* **2007**, *189*, 6176–6184.

(S25) Ganapathy, S.; Oostergetel, G. T.; Wawrzyniak, P. K.; Reus, M.; Chew, A. G. M.; Buda, F.; Boekema, E. J.; Bryant, D. A.; Holzwarth, A. R.; De Groot, H. J. Alternating syn-anti bacteriochlorophylls form concentric helical nanotubes in chlorosomes. *Proceedings of the National Academy of Sciences* **2009**, *106*, 8525–8530.

(S26) Günther, L. M.; Jendrny, M.; Bloemsma, E. A.; Tank, M.; Oostergetel, G. T.; Bryant, D. A.; Knoester, J.; Köhler, J. Structure of light-harvesting aggregates in individual chlorosomes. *The Journal of Physical Chemistry B* **2016**, *120*, 5367–5376.
